# A Quantum Chemical and Statistical Study of Cytotoxic Activity of Compounds Isolated from *Curcuma zedoaria*

**DOI:** 10.3390/ijms16059450

**Published:** 2015-04-27

**Authors:** Omer Abdalla Ahmed Hamdi, El Hassane Anouar, Jamil A. Shilpi, Zuhra Bashir Khalifa Al Trabolsy, Sharifuddin Bin Md Zain, Nur Shahidatul Shida Zakaria, Mohd Zulkefeli, Jean-Frédéric F. Weber, Sri Nurestri A. Malek, Syarifah Nur Syed Abdul Rahman, Khalijah Awang

**Affiliations:** 1 Department of Chemistry, Faculty of Science, University of Malaya, Kuala Lumpur 50603, Malaysia; E-Mails: omerhamdi2001@hotmail.com (O.A.A.H.); smzain@um.edu.my (S.B.M.Z.); 2 Department of Chemistry, College of Science, King Saud University, P.O. Box 2455, Riyadh 11451, Saudi Arabia; 3 Atta-ur-Rahman Institute for Natural Product Discovery, Universiti Teknologi MARA Kampus Puncak Alam, Bandar Puncak Alam 42300, Malaysia; E-Mails: zuhrabasher@yahoo.com (Z.B.K.A.T.); nursha8760@puncakalam.uitm.edu.my (N.S.S.Z.); mzmj@salam.uitm.edu.my (M.Z.); jffweber@puncakalam.uitm.edu.my (J.-F.F.W.); 4 Centre for Natural Products and Drug Discovery (CENAR), University of Malaya, Kuala Lumpur 50603, Malaysia; E-Mail: jamilshilpi@yahoo.com; 5 Institue of Biological Sciences, Faculty of Science, University of Malaya, Kuala Lumpur 50603, Malaysia; E-Mails: srimalek@um.edu.my (S.N.A.M.); snsar_attas@yahoo.com (S.N.S.A.R.)

**Keywords:** *Curcuma zedoaria*, diterpenes, sesquiterpenes, cytotoxicity, DFT, QSAR

## Abstract

A series of 21 compounds isolated from *Curcuma zedoaria* was subjected to cytotoxicity test against MCF7; Ca Ski; PC3 and HT-29 cancer cell lines; and a normal HUVEC cell line. To rationalize the structure–activity relationships of the isolated compounds; a set of electronic; steric and hydrophobic descriptors were calculated using density functional theory (DFT) method. Statistical analyses were carried out using simple and multiple linear regressions (SLR; MLR); principal component analysis (PCA); and hierarchical cluster analysis (HCA). SLR analyses showed that the cytotoxicity of the isolated compounds against a given cell line depend on certain descriptors; and the corresponding correlation coefficients (R^2^) vary from 0%–55%. MLR results revealed that the best models can be achieved with a limited number of specific descriptors applicable for compounds having a similar basic skeleton. Based on PCA; HCA and MLR analyses; active compounds were classified into subgroups; which was in agreement with the cell based cytotoxicity assay.

## 1. Introduction

*Curcuma zedoaria* (Christm.) Rosc. (Zingiberaceae) is a medicinal herb largely found in tropical Asian countries, including Malaysia, Indonesia, India, Japan and Thailand [1]. Also known as *temu putih* in Malaysia and Indonesia, *C. zedoaria* is widely consumed as spice, a flavouring agent in native dishes and is frequently used in food preparations for women during post-partum confinement [2,3]. It has long been used as a folk medicine in different Asian countries for the treatment of menstrual disorders, dyspepsia, vomiting, cancer, stomachic, blood stagnation, hepato-protection and for promoting menstruation [1,4,5]. The rhizomes of *C. zedoaria* is considered as a rich source of terpenoids [6].

Quantaum chemical methods can be successfully applied to express molecular interactions between substrate and receptor in terms of molecular electronic properties of the substrates. Various qualitative and quantitative analyses and relationship studies can be found in the literature that used quantum chemical and statistical methods to achieve correlations between calculated variables and biological activities of natural and synthetic substrates [7,8,9,10,11,12,13]. Ishihara *et al.* employed semi-empirical PM5 method to delineate the relationship between the cytotoxic activity and 11 chemical descriptors of a series of tropolone compounds and were able to show that the observed cytotoxic activity correlated well with compounds of structural similarities and was governed mainly by dipole moment (µ), hydrophobicity (logP), hardness (η), electrophilicity (ω) and electronegativity (χ) [14]. In another study, Stanchev *et al.* showed that the cytotoxic activity of a series of 4-hydroxycoumarins was well correlated with logP, µ, volume (V) and molecular orbital energies (E_HOMO_ and E_LUMO_) [15]. Yang *et al.* used a semi-empirical method AM1 to determine the molecular descriptors of a series of ganoderic acids with cytotoxicity against tumour cells; they showed that E_HOMO_, electronegativity, electronic energy, logP and molecular area (A) are the variables that best discriminate between highly and less active ganoderic acids [16].

**Figure 1:**
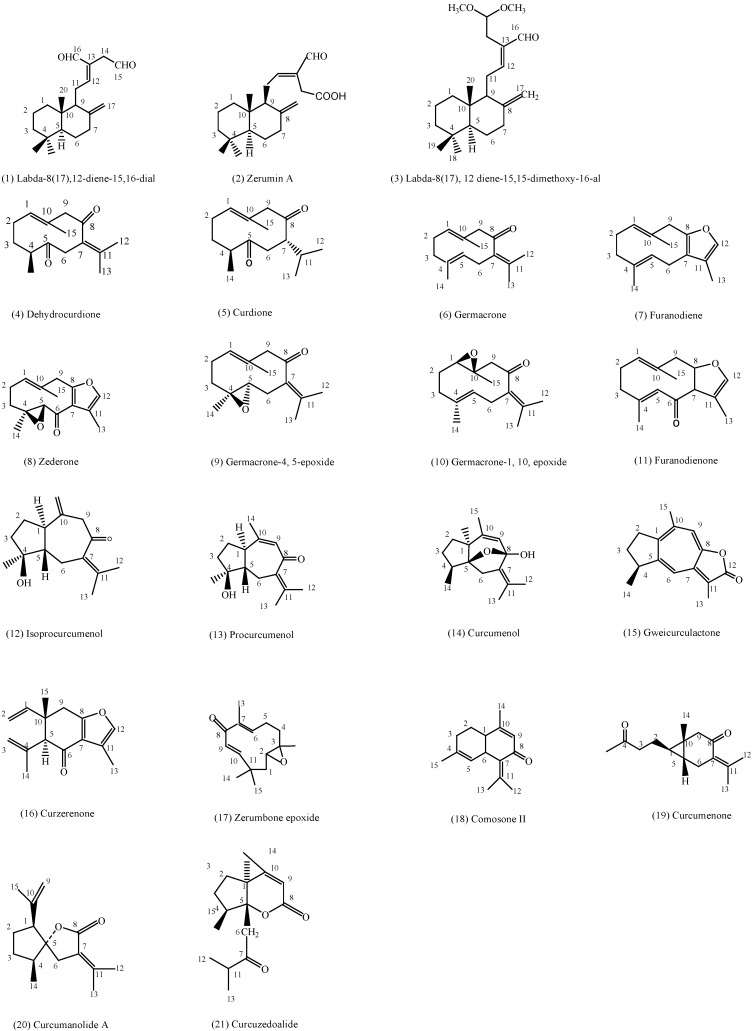
Structure of compounds isolated from *C. zedoaria*.

The present study aimed at elucidating the structure–cytotoxic activity relationships of a series of 21 compounds isolated from *C. zedoaria* (Figure 1) against four human cancer cells and a normal cell, namely as hormone-dependent breast carcinoma cells (MCF-7), cervical carcinoma cells (Ca Ski), human prostate cancer cells (PC-3), human colon adenocarcinoma cells (HT-29), and human umbilical vein endothelial cells (HUVEC). Density functional theory was adopted at the level of B3LYP/6-31+G (d, p) in order to calculate electronic and steric molecular descriptors of the isolated compounds, followed by the application of statistical methods (SLR, MLR, PCA and HCA) to determine the main descriptors responsible for the cytotoxic activity of the compounds under investigation.

## 2. Results and Discussion

### 2.1. Simple Linear Regression (SLR) Analysis

**Figure 2:**
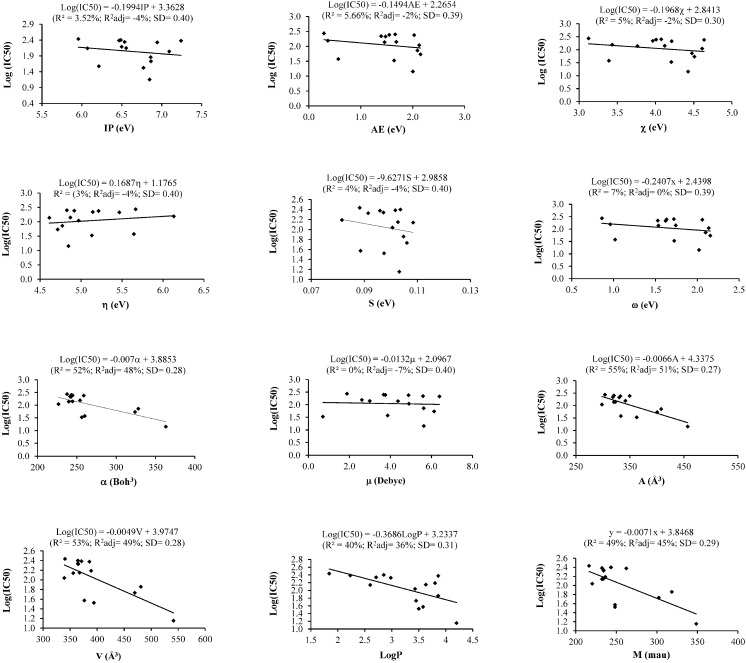
Simple linear regression correlation (SLR) curves between the cytotoxic activity on MCF-7 cells and each descriptor of isolated compounds from *C. zedoaria*.

The values of the electronic, steric and hydrophobic descriptors of compounds **1**–**21**, as well as their cytotoxic activities (IC_50_ in µM) against MCF-7, Ca Ski, PC-3, HT-29 and HUVEC cells are presented in Table 1. As observed, the cytotoxic activity of compounds (**1**–**21**) varied with the cell type. Thus, a simple linear regression analyses was done to determine the effect of each of the descriptors separately on the cytotoxicity of the isolated compounds. Figure 2 displays simple linear regression curves obtained with each descriptor for the cytotoxicity of the test compounds against MCF-7 cells, while the statistical parameters (correlation coefficient (R^2^), adjusted correlation coefficient (R^2^ adj) and standard deviation (SD)) for SLR curves between each descriptor and each tested cell line is presented in Table 2.

#### 2.1.1. Cytotoxicity against MCF-7 Cells and SLR Analysis

Based on the IC_50_ values (Table 2), the compounds were sorted into an inactive group (IC_50_ > 400 µM) and an active group (IC_50_ < 400 µM). To avoid the large discrepancies in the IC_50_ values, the active group was further subdivided into group A (200 < IC_50_ < 300 µM); group B (100 < IC_50_ < 200 µM) and group C (IC_50_ < 100 µM). The SLR analysis shows that the influence of a given descriptor on cytotoxic activity is dependent on the nature of the descriptor itself. For instance, the electronic descriptors IP, AE, χ, η, S, ω and μ have no significant influence (R^2^ ≈ 0%–7%), while modest correlations were observed for descriptors α, A, V, LogP and M (R^2^ ≈ 40%–55%) (Figure 2). These results are consistent with those obtained by Ishihara *et al.*, who showed that cytotoxic activity of 20 synthesized tropolones was poorly correlated with each of 11 chosen descriptors [14,17].

#### 2.1.2. Cytotoxicity against Ca Ski Cells and SLR Analysis

Following the same pattern as that of Section 2.1.1, the compounds were divided into inactive and active groups, while the active group was further subdivided into group A, group B, and group C based on their IC_50_ values against Ca Ski cells. Table 2 represents the SLR parameters between each descriptor and log(IC_50_). As it can be seen, the effects of electronic descriptors IP, AE, χ, η, S and ω are negligible (R^2^ ≤ 10%), while α, A, V and M descriptors showed a moderate effect (R^2^ ≈ 28%–43%). Surprisingly, hydrophobicity played no role on the cytotoxicity of the compounds (R^2^ ≈ 0) (Table 2).

**Table 1:** Cytotoxicity IC_50_ (µM) and molecular descriptors obtained at B3P86/6-311+G (d, p) level for the isolated compounds.

NO.	IP	EA	χ	η	ω	α	μ	A	V	Log P	M	IC_50_ (µM) ^a^				
												MCF-7	Ca Ski	PC-3	HT-29	HUVEC
1	6.87	2.15	4.51	4.71	2.16	324	6.13	400	470	3.45	302.46	53.9 ± 0.7	47.9 ± 0.3	87.0 ± 7.9	71.1 ± 10.2	149.8 ± 6.3
2	6.86	2.09	4.48	4.77	2.10	328	5.62	408	481	3.86	318.46	70.0 ± 3.3	NA ^b^	68.8 ± 5.0	54.6 ± 6.3	81.0 ± 6.0
3	6.85	2.00	4.43	4.85	2.02	363	5.63	457	541	4.20	348.53	14.3 ± 0.6	NA ^b^	119.6 ± 9.8	138.6 ± 14.6	135.7 ± 12.1
4	6.56	1.69	4.12	4.87	1.74	244	2.99	323	368	3.63	234.34	140.8 ± 4.7	92.6 ± 4.7	81.5 ± 11.9	96.9 ± 10.2	102.4 ± 9.0
5	6.52	1.26	3.89	5.26	1.44	238	1.40	331	377	4.01	236.35	NA ^b^	–	–	–	–
6	6.40	1.39	3.89	5.01	1.51	248	4.06	321	355	3.81	218.34	NA ^b^	180.0 ± 5.5	252.8 ± 22.4	196.5 ± 18.8	337.5 ± 1.4
7	5.96	0.29	3.12	5.67	0.86	238	1.88	304	341	1.84	216.32	271.9 ± 12.0	NA ^b^	182.6 ± 20.8	218.2 ± 20.3	189.1 ± 12.0
8	6.58	1.80	4.19	4.78	1.84	241	6.35	313	353	0.84	246.31	NA ^b^	NA ^b^	109.6 ± 7.7	77.5 ± 10.1	170.9 ± 11.0
9	6.54	1.39	3.96	5.14	1.53	241	5.59	318	364	2.71	234.34	218.8 ± 17.1	NA ^b^	187.3 ± 30.7	169.0 ± 19.6	206.5 ± 20.1
10	6.49	1.66	4.08	4.83	1.72	243	3.67	320	364	2.85	243.34	251.7 ± 23.9	NA ^b^	218.6 ± 20.1	299.2 ± 34.1	228.1 ± 6.6
11	6.07	1.46	3.76	4.61	1.54	240	4.37	320	356	2.60	232.32	137.7 ± 6.5	–	–	–	–
12	6.50	0.37	3.43	6.13	0.96	254	2.61	342	389	3.80	236.4	154.5 ± 17.8	NA ^b^	158.2 ± 19.0	218.3 ± 16.5	190.8 ± 12.7
13	6.54	1.77	4.16	4.78	1.81	252	7.48	327	362	2.71	234.34	127.2 ±9.4	266.3 ± 1.3	56.8 ± 7.3	66.1 ± 9.8	69.6 ± 4.3
14	6.21	0.57	3.39	5.64	1.02	260	3.86	333	376	3.58	248.37	37.4 ± 37.4	74.5 ± 4.0	69.7 ± 4.8	99.9 ± 10.9	104.3 ± 5.6
15	5.71	2.26	3.99	3.45	2.30	290	11.67	305	330	3.46	228.29	136.8 ± 14.1	NA ^b^	167.8 ± 9.6	156.4 ± 25.4	314.1 ± 26.7
16	6.47	1.55	4.01	4.92	1.63	245	3.76	350	371	2.23	232.32	243.2 ± 13.8	–	–	–	–
17	7.09	2.12	4.61	4.97	2.14	227	4.90	298	339	3.43	220.31	109.4 ± 0.5	156.6 ± 2.7	49.0 ± 8.6	62.2 ± 12.3	64.5 ± 5.0
18	6.51	1.80	4.15	4.71	1.83	247	6.66	310	340	3.51	216.32	NA ^b^	351.3 ± 5.5	NA ^b^	NA ^b^	NA ^b^
19	6.77	1.64	4.21	5.13	1.72	256	0.71	362	394	3.5	248.37	32.2 ± 4.0	NA ^b^	160.2 ± 16.9	174.3 ± 25.0	201.3 ± 34.6
20	6.94	1.48	4.21	5.47	1.62	243	6.39	330	365	2.98	234.34	212.4 ± 13.2	80.2 ± 10.2	90.9 ± 13.7	92.6 ± 29.9	–
21	7.24	2.03	4.63	5.21	2.06	259	4.88	332	386	3.86	262.35	238.0 ± 13.8	236.7 ± 30.9	221.8 ± 13.3	172.7 ± 29.7	–

^a^ The cytotoxicity results as reported by [18]; ^b^ NA = Not active.

**Table 2:** Correlation coefficients (R^2^), adjusted correlation coefficients (R^2^_adj_) and standard deviations (SD) of simple linear regression curves (SLR) between each descriptor and the tested cell lines.

Descriptor/s/SLR on Cells	MCF-7			Ca Ski			PC-3				HT-29				HUVEC		
	%R^2^	%R^2^_adj_	SD	%R^2^	%R^2^_adj_	SD	%R^2^	%R^2^_adj_	SD		%R^2^	%R^2^_adj_	SD		%R^2^	%R^2^_adj_	SD
IP	4	−4	0.40	0	−14	0.31	8	2	0.22		14	8	0.22		30	24	0.19
EA	6	−2	0.39	6	−8	0.30	5	−2	0.23		19	14	0.21		3	−4	0.23
χ	5	−2	0.39	2	−11	0.31	8	2	0.22		23	18	0.20		13	7	0.21
η	3	−4	0.40	11	−2	0.30	0	−6	0.23		5	−2	0.23		2	−6	0.23
S	4	−4	0.40	10	−3	0.30	0	−7	0.23		2	−5	0.23		5	−3	0.23
ω	7	0	0.39	4	−10	0.31	5	−1	0.23		14	19	0.21		3	−4	0.23
α	52	48	0.28	28	18	0.27	2	−5	0.23		5	−1	0.23		1	−7	0.23
DM	0	−7	0.40	9	−4	0.30	4	−3	0.23		13	7	0.22		0	−8	0.23
A	55	51	0.27	42	33	0.24	2	−4	0.23		4	−2	0.23		3	−4	0.23
V	53	49	0.28	43	34	0.24	3	−4	0.23		5	−1	0.23		5	−2	0.22
Log P	40	36	0.31	0	−14	0.31	−7	0	0.23		0	−6	0.23		1	−7	0.23
M	49	45	0.29	36	26	0.25	4	−3	0.23		8	2	0.22		−1	6	0.22

#### 2.1.3. Cytotoxicity against PC-3 Cells and SLR Analysis

The compounds were classified into groups on the basis of their activity against PC-3 cells in the same fashion as discussed earlier. All chosen descriptors showed negligible effect on cytotoxic activity (R^2^ ≈ 0%–8%) (Table 2).

#### 2.1.4. Cytotoxicity against HT-29 Cells and SLR Analysis

In case of cytotoxicity of the isolated compounds against HT-29 cells, moderate effects were obtained with the electronic descriptors namely IP, EA, χ, ω and μ with 14%, 19%, 23%, 14% and 13% correlation coefficients, respectively. In contrast to the results obtained for MCF-7 and Ca Ski cells, the steric descriptors did not show significant effects (R^2^ ≤ 8%) (Table 2).

#### 2.1.5. Cytotoxicity against HUVEC Cells and SLR Analysis

For HUVEC cells, all descriptors showed no significant effect on the cytotoxic activity (R^2^ ≤ 5%), except IP and χ, which showed moderate effects (30% and 13% correlation coefficients, respectively) (Table 2).

### 2.2. Multiple Linear Regression (MLR) Analysis

In an attempt to further investigate the correlations between the calculated descriptors and the cytotoxic activity of the isolated compounds against each cell line, MLR analysis was performed. MLR analysis was conducted only for the compounds of the active group.

#### 2.2.1. Cytotoxicity against MCF-7 Cells and MLR Analysis

**Figure 3:**
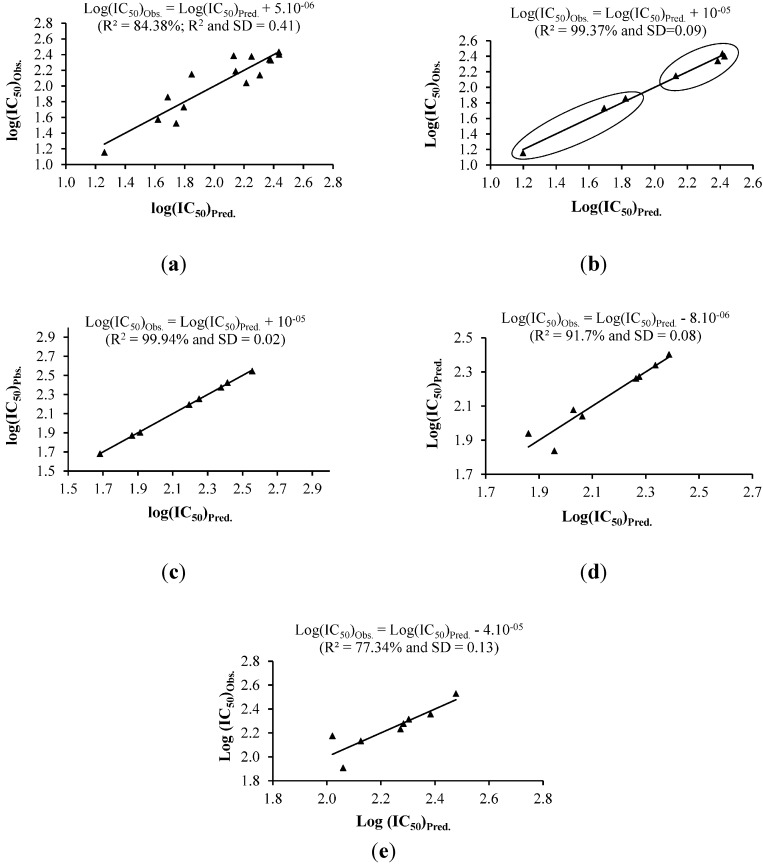
Multiple linear regression (MLR) correlations between most important descriptors and the cytotoxic activity of the active compounds isolated from *C. zedoaria*. (**a**) MLR analysis for cytotoxicity against MCF-7; (**b**) Modified MLR analysis of compounds **1**–**11** for cytotoxicity against MCF-7; (**c**) MLR analysis for cytotoxicity against Ca Ski; (**d**) MLR analysis for cytotoxicity against PC-3; (**e**) MLR analysis for cytotoxicity against HUVEC.

Among the 17 compounds for which the IC_50_ values were observed against MCF-7 cells, compound **15** (gweicurculactone) was used as the model compound, and therefore excluded from MLR analysis. The MLR model as given in Equation (1) was obtained from the correlation observed between log(IC_50_) and the descriptors. The corresponding curve is presented in Figure 3a.

(1) $$\text{log}{\left({\text{IC}}_{50}\right)}_{\text{Pred.}}=\text{−}(60.85\pm 73.65)+(2.91\pm 3.91)\text{IP}-(0.70\pm 2.22)\text{EA}+(1.10\pm 2.62)\text{χ}+(3.62\pm 3.60)\text{η}+(314.17\pm 359.28)\text{S}-(3.38\pm 9.43)\text{ω}-(0.01\pm 0.06)\text{α}-(0.05\pm 0.16)\text{μ}-(0.02\pm 0.03)\text{A}+(0.01\pm 0.04)\text{V}-(0.63\pm 0.38)\text{LogP}+(0.01\pm 0.03)\text{M}$$

The predicted log(IC_50_)_Pred._ and residuals to experimental log(IC_50_)_Obs._ for the active compounds are given in Table 3. The correlation between all descriptors and cytotoxicity is relatively weak, with a standard deviation of SD = 0.41 and R^2^ = 84%. The predicted log(IC_50_)_Pred._ for the model compound tested (compound **15**) is relatively high (4.48) with a residual value of 2.34. While the predicted IC_50_ value suggested compound **15** is categorised in the inactive group, the observed IC_50_ dictates it to be an active compound. Consequently, this model (Equation (1)) was considered not suitable for cytotoxicity prediction. To obtain a better model, the first 11 compounds (**1**–**11**) with similar skeletons were chosen for MLR analysis. For better consistency in the analysis, they were further subdivided into labdane diterpenes (compounds **1**–**3**) and germacrane sesquiterpenes (compounds **4**–**11**). Only the active compounds were subjected to MLR as shown in Equation (2) while compound **11** was selected as a test model in this study.

(2) $$\text{log}{\left({\text{IC}}_{50}\right)}_{\text{Pred.}}=\text{−}(7.77\pm 4.74)-(0.18\pm 0.52)\text{IP}-(0.06\pm 0.05)\text{A}+(0.03\pm 0.04)\text{V}-(0.07\pm 0.15)\text{LogP}+(0.02\pm 0.01)\text{M}$$

The model of Equation (2) was found to be superior to the previous model (Equation (1)) with a correlation coefficient of 99.37% and a SD of 0.09. For purpose of validation, this model (Equation (2)), was applied for compound **11**. The predicted log(IC_50_)_Pred._ was found to be 2.10, with a difference of only 0.04 from the experimental value. The difference between the predicted and observed cytotoxicity is 13 µM. The MLR model of Equation (2) demonstrated the importance of IP, steric parameters (area and volume), hydrophobicity (logP) and molecular weight (M) on the cytotoxic activity of the test compounds. These results are in good agreement with previous studies where steric parameters and hydrophobicity were found to be the most important descriptors to classify compounds into high and low activities [14,16]. Thus, the MLR of Equation (2) subdivided the test compounds into high and low cytotoxicity (Figure 3b).

#### 2.2.2. Cytotoxicity against Ca Ski Cells and MLR Analysis

Nine compounds showing cytotoxic activity against Ca Ski cells were selected for this study. Compound **4** (dehydrocurdione) was chosen as model compound and thus excluded from MLR analysis. The MLR model obtained between log(IC_50_) and six best descriptors is given in Equation (3) and the corresponding regression curve is shown in Figure 3c.

(3) $$\text{log}{\left({\text{IC}}_{50}\right)}_{\text{Pred.}}=\left(122.80\pm 7.42\right)-(11.88\pm 0.73)\text{η}-(608.30\pm 38.85)\text{S}+0.03\text{α}+0.02\text{A}-0.06\text{V}+0.03\text{M}$$

The predicted log(IC_50_) and residuals with respect to experimental values of active isolated compounds are presented in Table 3. The model was found to correlate the descriptors to the observed log(IC_50_) with good accuracy (R^2^ 99.94% and SD 0.02). For the model compound (**4**), the predicted log(IC_50_)_pred._ is 1.93, with a difference of 0.04 from the experimental value. The difference between the predicted and observed cytotoxicity is 8 µM. Equation (3) shows the function of steric parameters (area and volume), molecular weight (M), hardness, softness and the polarizability of the isolated compounds towards the cytotoxicity against Ca Ski cells.

#### 2.2.3. Cytotoxicity against PC-3 Cells and MLR Analysis

Seventeen compounds showing cytotoxicity against PC-3 cells were included in MLR analysis while compound **4** (dehydrocurdione) was excluded as the model compound. The MLR model (Equation (4)) obtained between log(IC_50_) and all descriptors gives a correlation of 88% (SD 0.17).

(4) $$\text{log}{\left({\text{IC}}_{50}\right)}_{\text{Pred.}}=\text{−}(42.62\pm 12.74)+(3.84\pm 0.94)\text{IP}+(217.76\pm 58.86)\text{EA}-(11.07\pm 2.96)\text{χ}-(0.20\pm 0.05)\text{η}+(2.98\pm 0.82)\text{S}-(0.23\pm 0.11)\text{ω}-(0.04\pm 0.01)\text{α}+(0.04\pm 0.02)\text{μ}+(0.02\pm 0.01)\text{A}-(0.01\pm 0.01)\text{V}+(0.98\pm 0.48)\text{LogP}+(2.49\pm 0.75)\text{M}$$

**Table 3:** Predicted log(IC_50_)_Pred_. and residuals of the active compounds obtained using MLR Equations (1)–(6).

NO.	Equation (1)		Equation (2)		Equation (3)		Equation (5)		Equation (6)	
	log(IC_50_)_Pred._	Resid.	log(IC_50_)_Pred._	Resid.	log(IC_50_)_Pred._	Resid.	log(IC_50_)_Pred._	Resid.	log(IC_50_)_Pred._	Resid.
1	1.79	0.06	1.73	1.69	1.68	0.00	1.86	−0.08	2.02	2.18
2	1.69	−0.17	1.86	1.82	–	–	1.96	0.12	2.06	1.91
3	1.26	0.11	1.15	1.20	–	–	2.03	−0.05	2.13	2.13
4	1.85	−0.30	2.15	2.13	–	–	2.39	−0.02	–	–
5	–	–	–	–	–	–	2.26	0.00	–	–
6	–	–	–	–	2.26	0.00	2.06	0.02	2.48	2.53
7	2.43	0.00	2.43	2.41	–	–	2.28	0.00	2.28	2.28
8	–	–	–	–	–	–	2.34	0.00	2.27	2.23
9	2.37	0.03	2.34	2.39	–	–	–	–	2.30	2.32
10	2.44	0.04	2.40	2.43	–	–	–	–	2.38	2.36
11	2.31	0.17	–	–	–	–	–	–	–	–
12	2.14	−0.05	–	–	–	–	–	–	–	–
13	–	–	–	–	2.43	-0.01	–	–	–	–
14	1.62	0.05	–	–	1.87	-0.01	–	–	–	–
15	–	–	–	–	–	–	–	–	–	–
16	2.13	−0.26	–	–	–	–	–	–	–	–
17	2.22	0.18	–	–	2.19	0.00	–	–	–	–
18	–	–	–	–	2.55	0.01	–	–	–	–
19	1.74	0.22	–	–	–	–	–	–	–	–
20	2.38	0.05	–	–	1.90	0.01	–	–	–	–
21	2.25	−0.13	–	–	2.37	0.00	–	–	–	–

The predicted IC_50_ (>400 µM) of compound (**4**) suggested it is an inactive compound, which is contradictory to the observed IC_50_ (81.5 µM) against PC-3 cells. In an attempt to derive a better model, the number of descriptors was reduced and the analysis was confined to compounds (**1**–**11**) with a similar basic skeleton. Compound **4** was excluded from MLR analysis. The best correlation was obtained with the electronic descriptors of IP, EA, ω and µ (Equation (5) and Figure 3d). The predicted log(IC_50_) and residuals to experimental results are presented in Table 3.

(5) $$\text{log}{\left({\text{IC}}_{50}\right)}_{\text{Pred.}}=\left(2.52\pm 3.16\right)+(0.36\pm 0.61)\text{IP}+(2.09\pm 0.54)\text{EA}-(3.30\pm 0.92)\text{ω}-(0.08\pm 0.05)\text{µ}$$

The predicted log(IC_50_)_Pred._ for the test compound **4** is 1.91, with a difference of 0.48 from the experimental value. Although the difference between the predicted (244 µM) and experimental IC_50_ value (82 µM) is relatively high (162 µM), MLR analysis categorised it in the active group which is consistent with the observed results against PC-3 cells.

#### 2.2.4. Cytotoxicity against HT-29 Cells and MLR Analysis 

Seventeen compounds showing cytotoxicity against HT-29 cells (Table 1) were chosen for this study while compound **4** (dehydrocurdione) was excluded from MLR analysis. The MLR model obtained between log(IC_50_) and all descriptors derived a correlation of 81% (SD 0.22). The predicted value for the test compound **4** (IC_50_ = 283 µM) suggested it as an active compound (experimental IC_50_ = 97 µM). In terms of activity, compound **4** falls in group A as per predicted IC_50_, which is quite different from its group (C) determined from the experimental IC_50_. In an attempt to obtain a better model, the number of descriptors was reduced and the analysis was performed for the first 11 compounds (**1**–**11**) with similar basic skeleton and compound **4** was excluded from MLR analysis. However, in every case, the difference between experimental and predicted IC_50_ was more than 100 µM, and therefore not presented herein.

#### 2.2.5. Cytotoxicity against HUVEC Cells and MLR Analysis

Fifteen compounds that showed activity against HUVEC cells were included in this study and compound **4** (dehydrocurdione) was excluded from MLR analysis. The correlation between log(IC_50_) and all descriptors gave an R^2^ of 96% (SD 0.16). The predicted IC_50_ for compound **4** from this model was 142 µM higher than the experimental IC_50_. The best correlation was obtained when three descriptors, namely IP, χ, S and V were taken into consideration (R^2^ 77%, SD 0.13) (Equation (6)). Predicted IC_50_ of compound **4** classified it as an active compound with a difference of 48 µM from the experimental IC_50_ value.

(6) $$\text{log}{\left({\text{IC}}_{50}\right)}_{\text{Pred.}}=\left(72.92\pm 37.80\right)-(14.02\pm 7.48)\text{IP}+(13.07\pm 7.14)\text{χ}+(13.07\pm 7.14)\text{S}-(0.0004\pm 0.0016)\text{V}$$

### 2.3. Principal Component Analysis (PCA)

**Figure 4:**
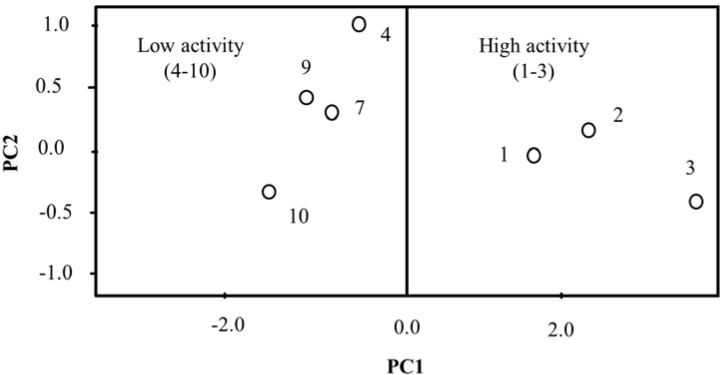
Plot of the score vectors of first principal components for cytotoxicity of compounds from *C. zedoaria* against MCF-7 cells.

Principal component analysis (PCA) allows the reduction of the number of variables used in a statistical analysis to create a new set of variables (PCs) expressed in a linear combination of the original data set [19]. The first new variable (PC1) contains the largest variance; while the second contains the second largest variance, and so on. Before applying the PCA method, each variable was standardized for ease of comparison between each other on the same scale. PCA analysis was performed only on MCF-7 cells. After several attempts to obtain a good classification of the isolated compounds, the best result was achieved with five variables, namely IP, A, V, logP and M. The first three components of PCA (PC1 = 90.50%, PC2 = 7.12%, and PC3 = 2.27%) conceded 99.89% of the overall variance of the data set (Table 4), while the sole combination of PC1 and PC2 described 97.62% of variance (Table 4). The loading vectors for PC1, PC2, and PC3 are given in Table 5 and the plot of the score vectors of the two principal components (PC1 × PC2) is shown in Figure 4.

**Table 4:** Variances (eigenvalues) obtained for the first three principal components.

Component	Eigenvalue	Variance (%)	Cumulated Variance (%)
PC1	4.525	90.50	90.50
PC2	0.3560	7.12	97.62
PC3	0.1134	2.27	99.89

**Table 5:** Loading vectors for the first three principal components.

Variable	PC1	PC2	PC3
IP	0.43	0.61	0.66
A	0.46	−0.38	−0.04
V	0.46	−0.34	0.04
logP	0.43	0.51	−0.74
M	0.46	−0.32	0.08

As can be seen in Figure 4, the compounds under investigation are divided into two groups based on PCA analysis: compounds with high activity (**1**–**3**) and low activity (**4**–**10**). The principal component PC1 presented in Table 5 can be expressed through the following equation:

(7) PC1=0.34IP+0.46EA+0.46V+0.43logP+0.46M

Thus, a compound can be considered active if its IP, A, V, log P, and M values are similar to those described in the above Equation (7). When compared with published literature, the results of our present investigation followed the same trend with some agreement and disagreement in the involvement of descriptors for the activity of a series of compounds. For example, Yang *et al.* showed that cytotoxic ganoderic acids can be attained when higher values for the variables E_HOMO_, V, E_el_, and logP are coupled with a smaller value for M [16]. Sauza *et al.* found that for a given flavone to be active against HIV, it must have smaller values for log P and V while EA must have a larger value [7]. In the present study, the results obtained from MLR or PCA are in coordination to show that the cytotoxicity of the compounds under investigation is dependent on the same descriptors (IP, A, V, logP and M) and afforded the same classification of the compounds (Figure 3b and Figure 4).

### 2.4. Hierarchical Cluster Analysis (HCA)

**Figure 5:**
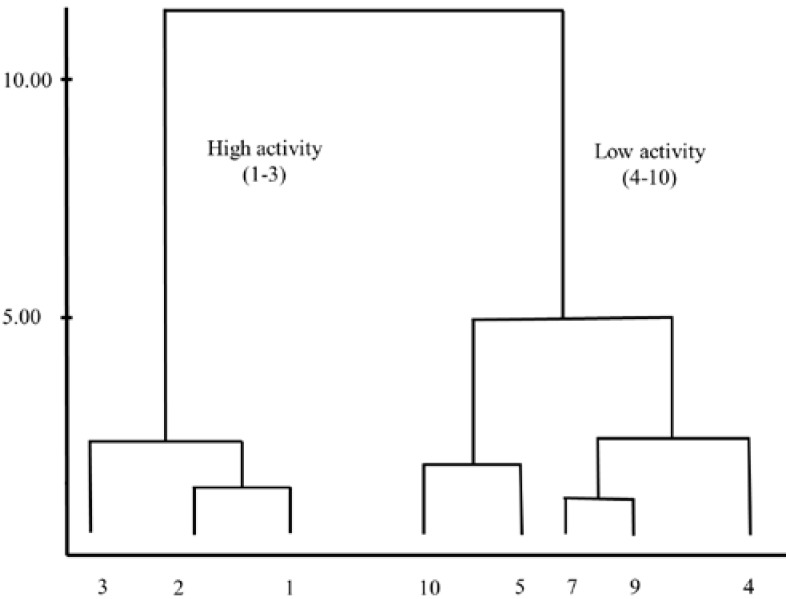
Dendrogram obtained from HCA of cytotoxicity of compounds from *C. zedoaria*.

In case of preliminary data analysis, HCA is a powerful tool for examining data sets for expected or unexpected clusters, including the presence of outliers. It examines the distances between the samples in a data set and represents them in a dendrogram which provides similar information as that of PCA results [20]. In HCA, each point forms only one cluster, and then the similarity matrix is analysed. The most similar points are assembled forming one cluster and the process is repeated until all the points belong to only one group [20]. The results obtained from MCF-7 cells are presented in the dendrogram (Figure 5). Vertical lines in the dendrogram represent the compounds while the horizontal lines represent the distances between compounds within the same group or from compounds of other groups. According to the distances, the compounds are subdivided into highly and weakly active groups and this classification is similar to those obtained from PCA and MLR analysis.

## 3. Experimental Section

### 3.1. Extraction and Isolation of Compounds from C. zedoaria

Characterization of the isolated compounds (Figure 1) from *C. zedoaria* was performed by extensive spectroscopic studies including 1D, 2D NMR spectroscopy, GC, GC-MS analysis, and compared with those reported in literature [21,22,23,24]. ^1^H and ^13^C NMR spectra of the isolated compounds (Figure 1) can be found in the supplementary data file. These compounds can be classified as labdane type diterpenes (**1**–**3**), germacrane type sesquiterpenes (**4**–**11**), guaiane type sesquiterpenes (**12**–**15**), elemane type sesquiterpenes (**16**), humulane type sesquiterpenes (**17**), cadinane type sesquiterpenes (**18**), carabrane type sesquiterpenes (**19**), spirolactone type sesquiterpenes (**20**), and a *seco*-guaiane type sesquiterpene (**21**).

### 3.2. Theoretical Calculations

Energy minimization and 3D structure optimization of the compounds were done by popular Becke three parameter Lee-Yang-Parr (B3LYP) exchange-correlation hybrid functional combined with a double-ζ Pople-type basis set 6-31+G (d,p), in which polarized and diffuse functions are taken into consideration [25]. B3LYP hybrid functional includes a mixture of Hartree-Fock exchange (20% of HF) with DFT exchange-correlation functional. The frequency analyses were carried out at the same level of theory. The absence of imaginary frequencies confirmed that the structures are true minima on the potential energy surface. The choice of the hybrid functional B3LYP is based on previous QSAR studies [26,27]. Recently, we successfully applied the hybrid functional B3P86 to calculate the electronic and structural descriptors for a series of phenolic Schiff bases [28].

The chemical descriptors selected to correlate with cytotoxic activity are: (i) electronic descriptors: frontier molecular orbital energies (E_HOMO_, E_LUMO_, which are well accepted as molecular descriptors in medicinal chemistry, since they are linked to the capacity of a molecule to form charge transfer complex with its biological receptor), ionization potential (IP), electronic affinity (EA), electronegativity (χ), hardness (η), softness (S), electrophilicity index (ω), dipole moment (μ), molecular polarizability (α); (ii) steric descriptors: surface area of a molecule (A), volume (V) and its molecular weight (M); and (iii) hydrophobicity descriptor: logP, where P stands for the octanol-water partition coefficient. The calculations of logP were carried out using Hyperchem Molecular package [29] by means of the atomic parameters derived by Ghose, Pritchett and Crippen and later extended by Ghose and co-workers [30,31]. The other descriptors were calculated using the DFT method and obtained in two different ways: (i) Orbital consideration, which is based on Koopman’s theorem where IP = −E_HOMO_ and EA = −E_LUMO_ [32]; and (ii) energy consideration, which is based on the use of the classical finite difference approximation, where the change of one electron is usually involved ΔN = ±1 [33]. In this method, IP = E_+1_ − E_0_ and EA = E_0_ − E_−1_ where E_0_, E_−1_ and E_+1_ are the electronic energies of neutral molecule, when adding and removing an electron to the neutral molecule, respectively. In addition to methods (i) and (ii), the electronic descriptors (e.g., hardness) can be calculated using internally resolved hardness tensor (IRHT) approach [34,35,36], which deals with the fractional occupation numbers based on Janak’s extension of DFT [37]. This approach is also based on orbital energies and takes into account the fractional occupation numbers based on Janak’s extension of DFT [37]. De Luca *et al.* used the above approaches to investigate the solvent effects on the hardness values of a series of neutral and charged molecules, and found that these three methods can give similar results in the presence of solvent [38].

The solvent effects were taken into account implicitly by using the polarizable continuum model (PCM) as implemented in the Gaussian 09 package [39]. In PCM, the solute is embedded into a cavity surrounded by solvent described by its dielectric constant ε (e.g., for methanol ε = 32.6) [40]. The use of an explicit solvent has been investigated notably by Guerra *et al.*, who obtained a better description of the electronic properties using PCM compared to the explicit solvent [41]. A hybrid model was tested by Trouillas *et al.* [42]. The authors showed that only slight differences can be observed as compared to PCM. All theoretical calculations including ground state geometry optimization and frequency analysis calculations were performed with Gaussian 09 package [39].

Simple and multiple linear regression (SLR and MLR, respectively) analyses were used to determine regression equations, correlation coefficients R^2^, adjusted R^2^ and standard deviations (SD). PCA and HCA were employed to reduce dimensionality and investigate the subset of descriptors that could be more effective for classifying the isolated compounds according to their degree of cytotoxicity against tumour cells.

The regression models and statistical analyses of obtained results were carried out by using DataLab package [43].

## 4. Conclusions

In the present study, a set of electronic, steric and hydrophobicity descriptors were analysed using DFT quantum chemical calculations for a series of 21 compounds from *C. zedoaria* to determine the effect of the descriptors towards their cytotoxic activity against four different types of cancer cells (MCF-7, Ca Ski, PC-3 and HT-29), as well as a normal cell line (HUVEC). The statistical analyses showed that the influence of individual descriptor on the cytotoxicity of these compounds is not significant with an R^2^ less than 50% and a standard deviation higher than 0.20. The results also showed that the cytotoxicity of the compounds towards a given cell line rather depends on a set of certain descriptors. MLR, PCA and HCA allowed us to define the cytotoxicity of the compounds as high, moderate, and low based on their cytotoxicity.

## Supplementary Materials

Supplementary materials can be found at http://www.mdpi.com/1422-0067/16/05/9450/s1.
