# Supplementary material for: A Quantum Chemical and Statistical Study of Cytotoxic Activity of Compounds Isolated from Curcuma zedoaria
Source: Int J Mol Sci. 2015 Apr 27;16(5):9450–68. doi: 10.3390/ijms16059450 (PMC4463598; doi:10.3390/ijms16059450)
Supplement: Supplementary file 1 [file ijms-16-09450-s001.pdf]

Supplementary Information

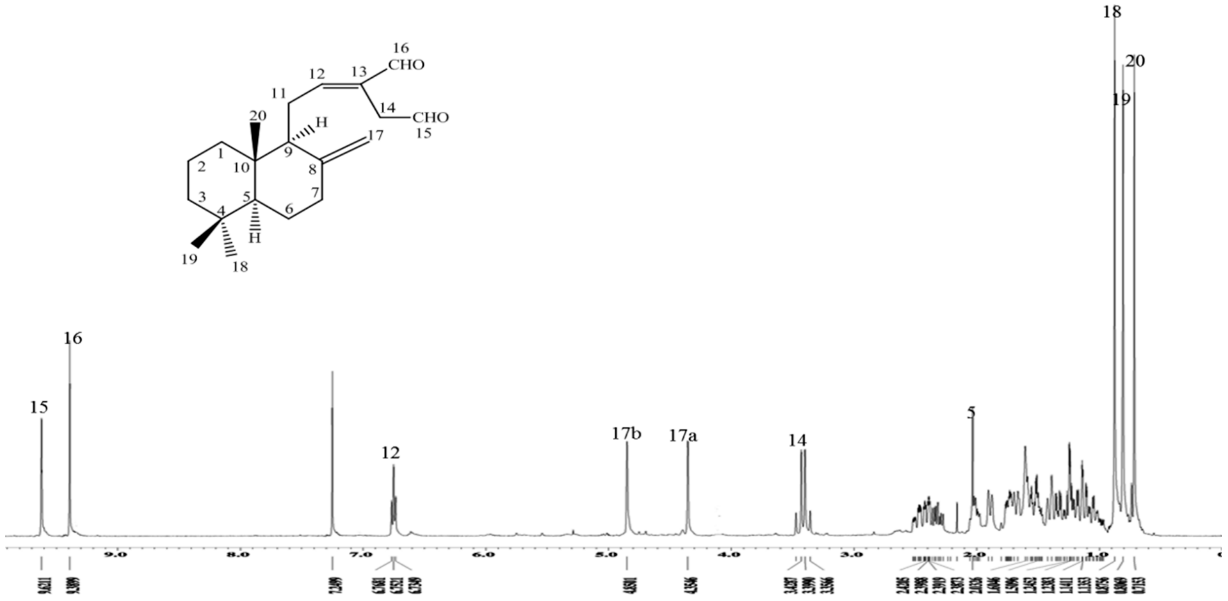

Figure S1. ^1H NMR spectrum of labda-8(17), 12-diene-15, 16-dial (**1**) in $CDCl_3$. labda-8(17),12-diene-15,16-dial (**1**): ^1H NMR ($CDCl_3$, 400 MHz), δ: 0.71 (3H, *s*, H-20), 0.80 (3H, *s*, H-19), 0.87 (3H, *s*, H-18), 4.37, 4.84 (2H, *br.s*, H-17), 9.38 (1H, *s*, H-16), 9.62 (1H, *s*, H-15), 3.36 (1H, *d*, J = 16.9 Hz, H-14), 6.75 (1H, *t*, J = 6.4 Hz, H-12), 2.34, 2.50 (2H, *m*, H-11), 1.92 (1H, *d*, J = 11.4 Hz, H-9) , 2.03, 2.42 (2H, *m*, H-7), 1.34, 1.76 (2H, *m*, H-6), 1.3 (1H, *dd*, J = 2.8, 12.8 Hz, H-5), 1.22, 1.42 (2H, *m*, H-3), 1.52, 1.58 (2H, *m*, H-2), 1.07, 1.72 (2H, *m*, H-1).

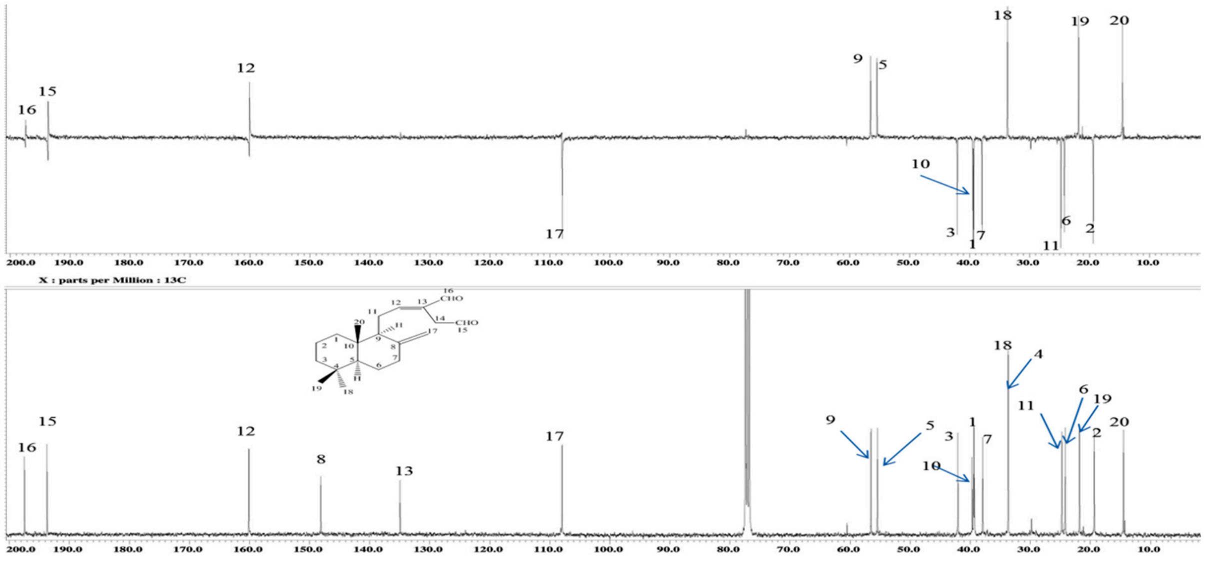

Figure S2. ^{13}C and DEPT spectra of labda-8(17), 12-diene-15, 16-dial (**1**) in $CDCl_3$. labda-8(17), 12-diene-15,16-dial (**1**): ^{13}C NMR ($CDCl_3$, 100 MHz), δ: 14.4 (C-20), 21.8 (C-19), 33.6 (C-18), 107.9 (C-17), 193.6 (C-16), 197.4 (C-15), 39.4 (C-14), 134.9 (C-13), 160.1 (C-12), 24.7 (C-11), 39.6 (C-10), 56.5 (C-9), 148.1 (C-8), 37.9 (C-7), 24.1 (C-6), 55.4 (C-5), 33.6 (C-4), 42.0 (C-3), 19.3 (C-2), 39.3 (C-1).

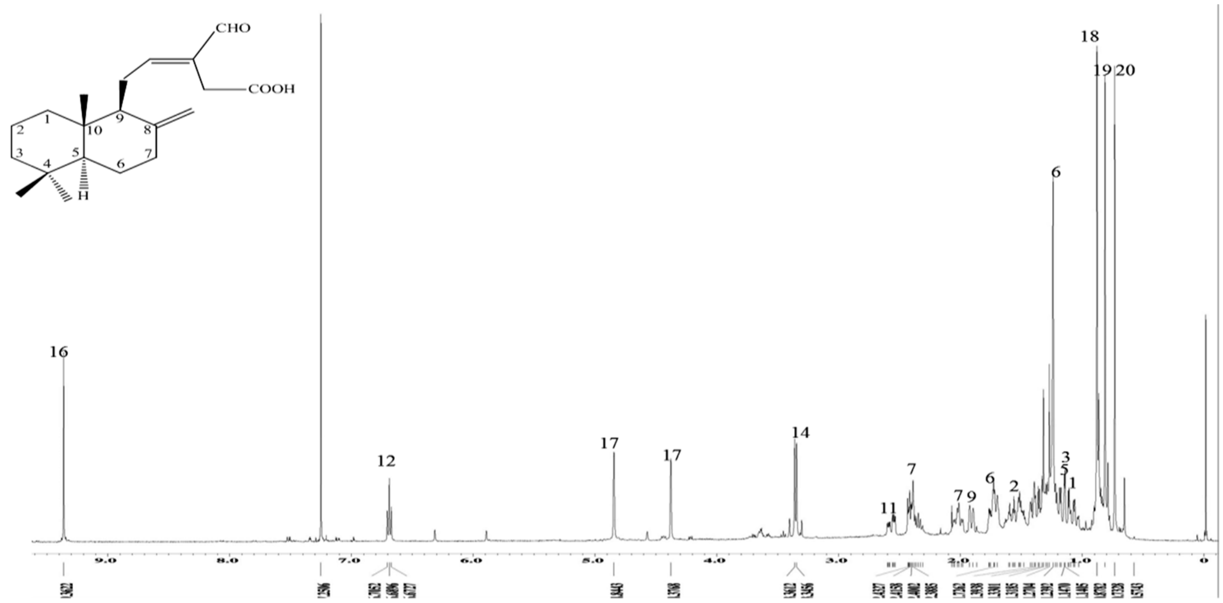

Figure S3. ^{1}H NMR spectrum of Zerumin A (**2**) in $CDCl_3$. Zerumin A (**2**): ^{1}H NMR ($CDCl_3$, 400 MHz), δ: 0.73 (3H, *s*, H-20), 0.81 (3H, *s*, H-19), 0.87 (3H, *s*, H-18), 4.37, 4.84 (2H, *br.s*, H-17), 9.37 (1H, *s*, H-16), 3.36 (1H, *d*, *J* = 16.4 Hz, H-14), 6.68 (1H, *t*, *J* = 6.4 Hz, H-12), 2.55 (2H, *ddd*, *J* = 2.7, 3.2, 6.3 Hz, H-11), 1.92 (1H, *d*, *J* = 11 Hz, H-9), 2.41 (1H, *m*, H_b-7), 2.02 (1H, *dd*, *J* = 5.0, 13.2 Hz, H_b-7), 1.36, 1.75 (2H, *m*, H-6), 1.13, 1.75 (1H, *dd*, *J* = 2.7, 12.8 Hz, H-5), 1.21, 1.42 (2H, *m*, H-3), 1.52, 1.59 (2H, *m*, H-2), 1.07, 1.72 (2H, *m*, H-1).

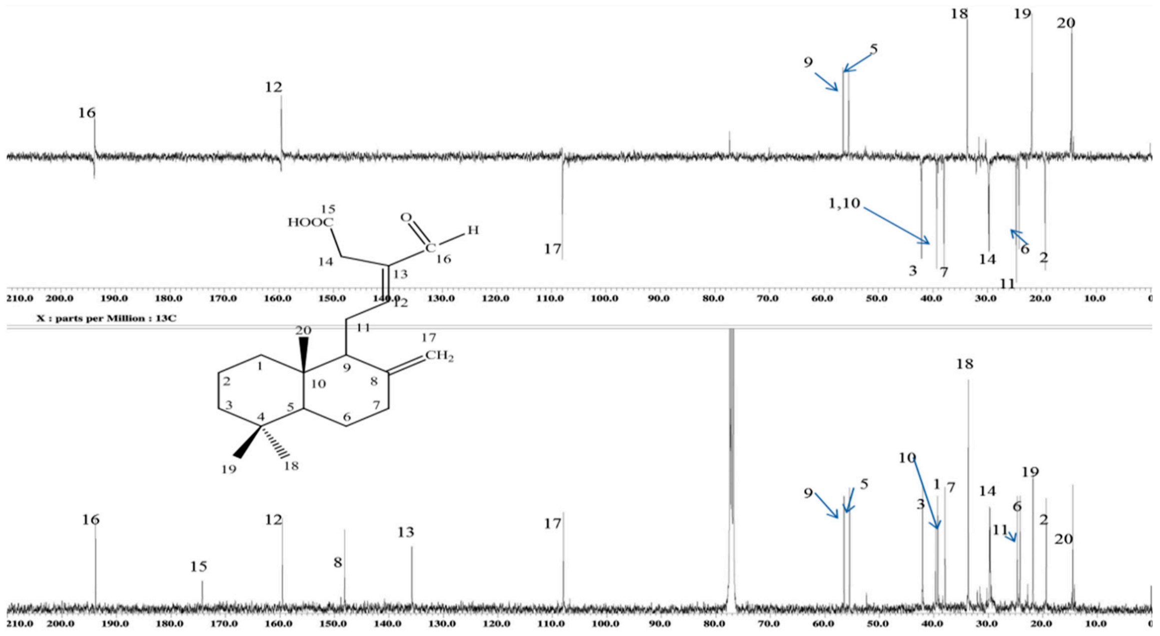

Figure S4. ^{13}C and DEPT spectra of Zerumin A (**2**) in $CDCl_3$. Zerumin A (**2**): ^{13}C NMR ($CDCl_3$, 100 MHz), δ: 14.1 (C-20), 21.8 (C-19), 33.6 (C-18), 107.9 (C-17), 193.8 (C-16), 174.3 (C-15), 29.7 (C-14), 135.7 (C-13), 159.5 (C-12), 24.6 (C-11), 39.6 (C-10), 56.4 (C-9), 148.1 (C-8), 37.9 (C-7), 24.1 (C-6), 55.4 (C-5), 33.6 (C-4), 42.0 (C-3), 19.3 (C-2), 39.2 (C-1).

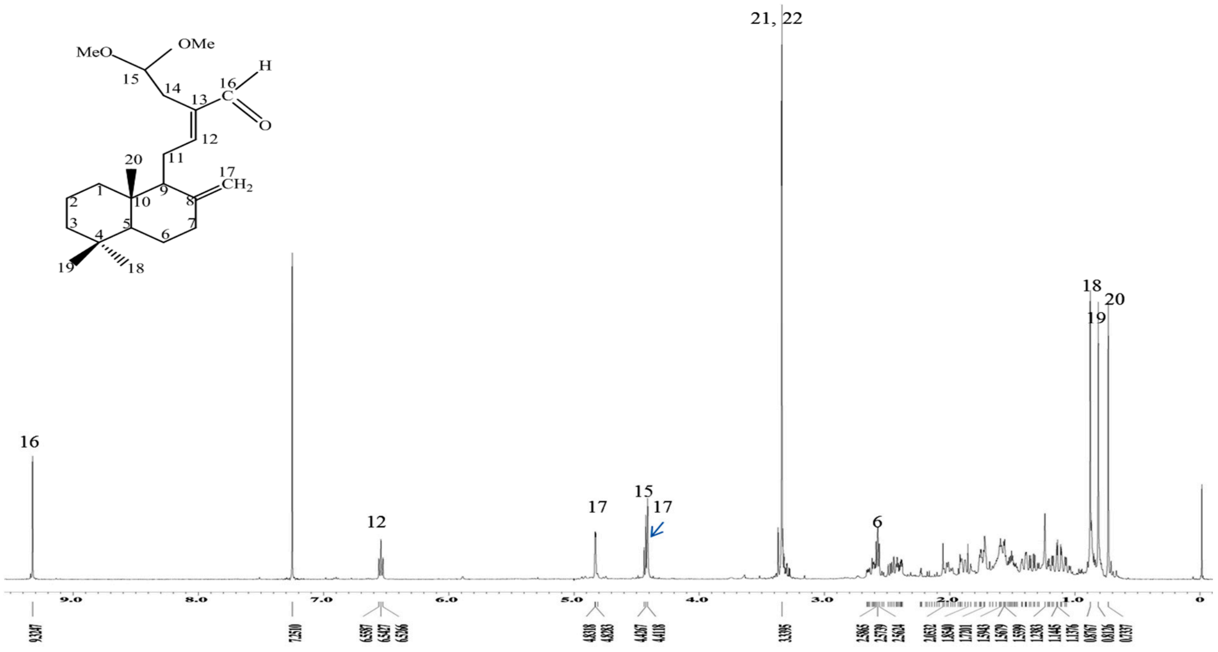

Figure S5. ^{1}H NMR spectrum of Calcaratarin A (**3**) in $CDCl_3$. Calcaratarin A (**3**): ^{1}H NMR ($CDCl_3$, 400 MHz), δ: 3.33 (2 OMe, *s*, H-21,22), 0.73 (3H, *s*, H-20), 0.81 (3H, *s*, H-19), 0.87 (3H, *s*, H-18), 4.41, 4.82 (2H, *dd*, *J* = 1.4, 0.48 Hz, H-17), 9.32 (1H, *s*, H-16), 4.41 (2H, *t*, *J* = 5.4 Hz, H-15), 2.56 (2H, m, H-14), 6.53 (1H, *t*, *J* = 5.9 Hz, H-12), 2.42, 2.62 (2H, *m*, H-11), 1.92 (1H, *d*, *J* = 11 Hz , H-9), 2.0, 2.38 (2H, *m*, H-7), 1.32, 1.72 (2H, *m*, H-6), 1.12 (1H, *dd*, *J* = 2.7, 12.8 Hz, H-5), 1.03, 1.2 (2H, *br.s*, *d*, *J* = 1.36 Hz, H-3), 1.47, 1.56 (2H, *br.s*, *m*, H-2), 1.07, 1.74, (2H, *m*, H-1).

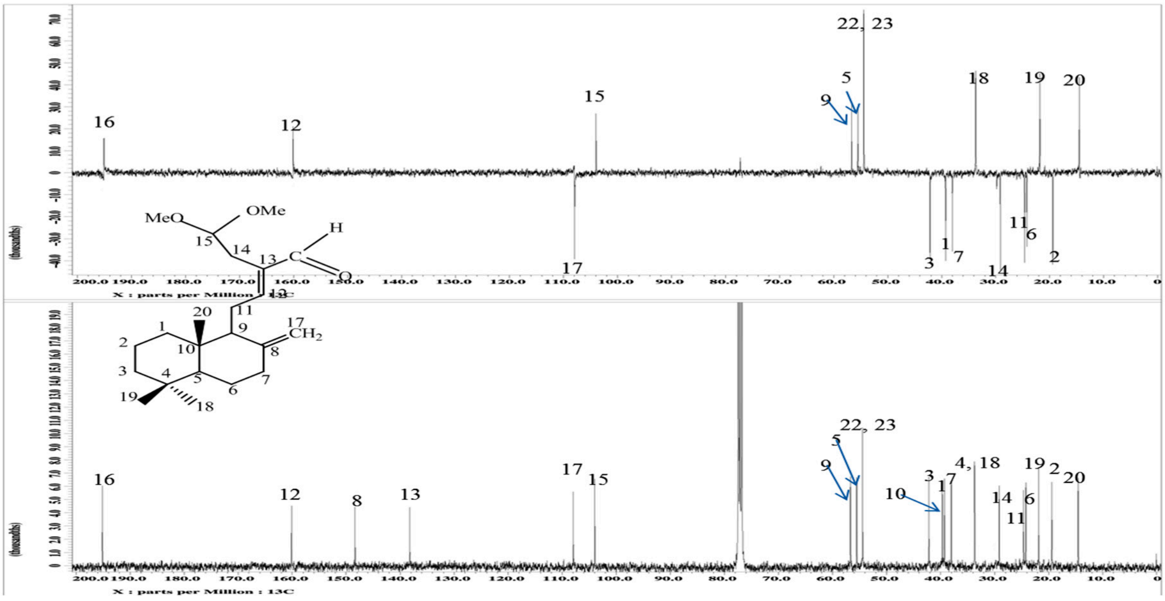

Figure S6. ^{13}C and DEPT spectra of Calcaratarin A (**3**) in $CDCl_3$. Calcaratarin A (**3**): ^{13}C NMR ($CDCl_3$, 100 MHz), δ: 54.4 (C-21, 22), 14.5 (C-20), 21.8 (C-19), 33.7 (C-18), 107.9 (C-17), 195.1 (C-16), 103.9 (C-15), 29.1 (C-14), 138.2 (C-13), 160.1 (C-12), 24.6 (C-11), 39.6 (C-10), 56.6 (C-9), 148.3 (C-8), 37.9 (C-7), 24.2 (C-6), 55.5 (C-5), 33.6 (C-4), 42.1 (C-3), 19.4 (C-2), 39.2 (C-1).

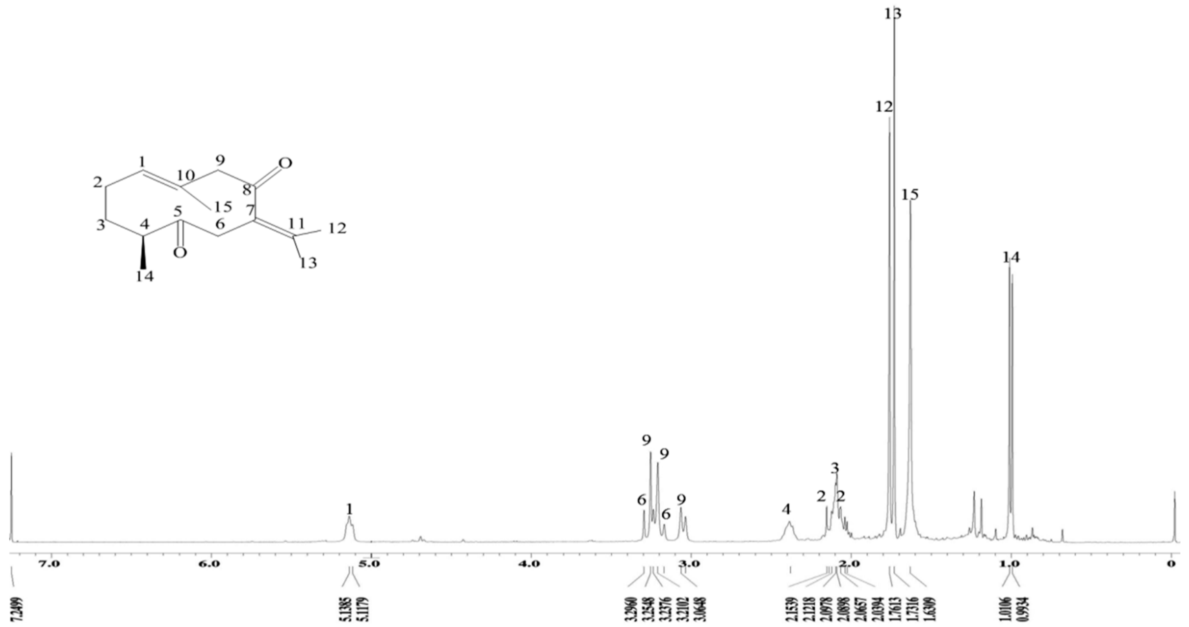

Figure S7. ^{1}H NMR spectrum of dehydrocurdione (**4**) in $CDCl_3$. Dehydrocurdione (**4**): ^{1}H NMR ($CDCl_3$, 400 MHz), δ: 1.63 (3H, *s*, H-15), 1.01 (3H, *d*, *J* = 6.88 Hz, H-14), 1.73 (3H, *s*, H-13), 1.76 (3H, *s*, H-12), 3.06, 3.23 (2H, *dd*, *J* = 11.44 Hz, H-9), 3.21, 3.29 (2H, *dd*, *J* = 16.48 Hz, H-6), 2.38 (1H, *m*, H-4), 2.0 (2H, *m*, H-3), 2.10 (2H, *m*, H-2). 5.13 (1H, *t*, *J* = 8.24 Hz, H-1).

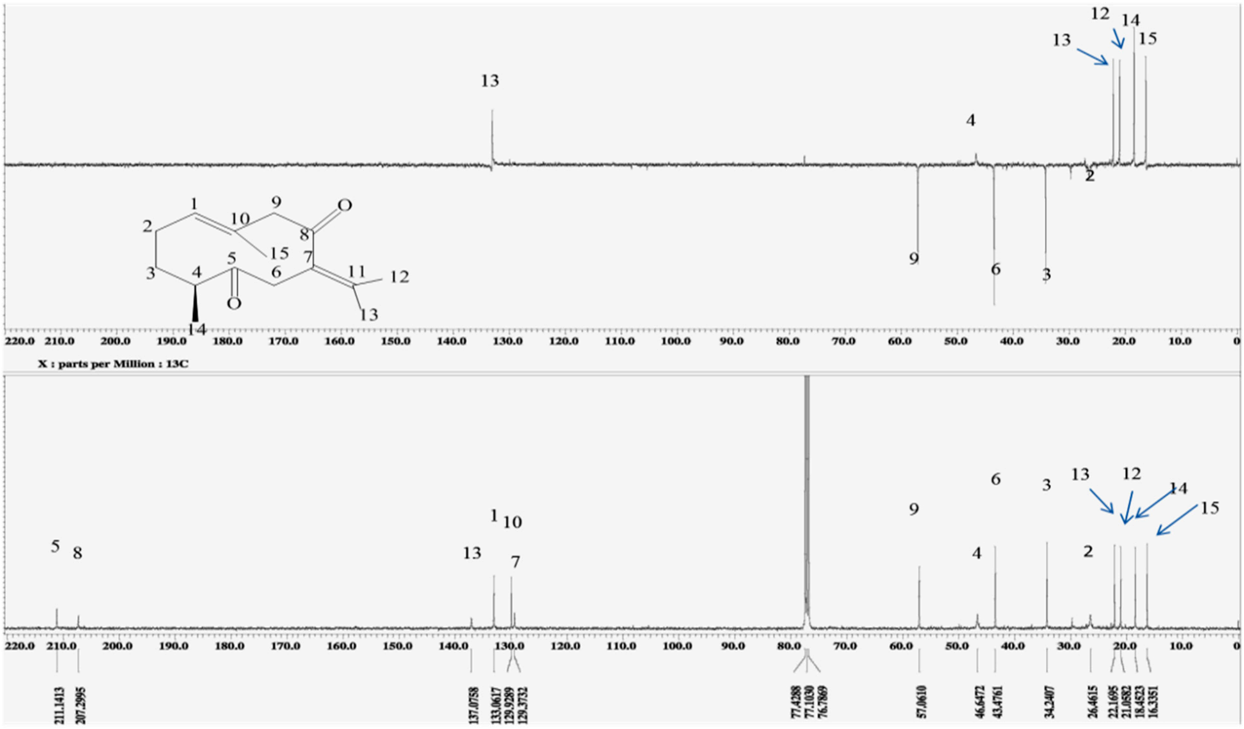

Figure S8. ^{13}C and DEPT spectra of dehydrocurdione (**4**) in $CDCl_3$. Dehydrocurdione (**4**): ^{13}C NMR ($CDCl_3$, 100 MHz), δ: 16.3 (C-15), 18.4 (C-14), 22.1 (C-13), 21.0 (C-12), 137.0 (C-11), 129.9 (C-10), 57.0 (C-9), 207.2 (C-8), 129.3 (C-7), 43.4 (C-6), 211.1 (C-5), 46.4 (C-4), 34.2 (C-3), 26.4 (C-2), 133.0 (C-1).

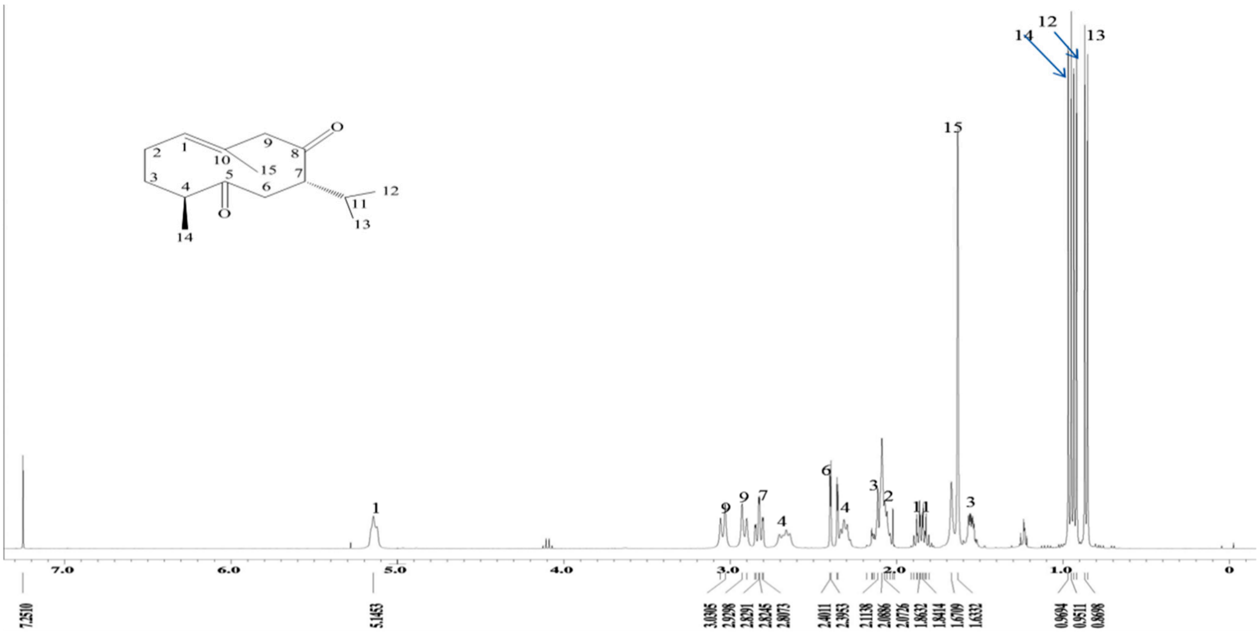

Figure S9. ^{1}H NMR spectrum of Curdione (**5**) in $CDCl_3$. Curdione (**5**): ^{1}H NMR ($CDCl_3$, 400 MHz), δ: 1.67 (3H, *s*, H-15), 0.96 (3H, *d*, *J* = 6.8 Hz, H-14), 0.88 (3H, *d*, *J* = 6.4 Hz, H-13), 0.93 (3H, *d*, *J* = 6.4 Hz, H-12), 1.84 (1H, *m*, H-11), 2.91, 3.04 (2H, *d*, *J* = 11 Hz, H-9), 2.81 (1H, *m*, H-7), 2.4 (2H, *m*, H-6), 2.36 (1 H, *m,* H-4), 1.63, 2.08 (2H, *m*, H-3), 2.08 (2H, *m*, H-2), 5.1 (1H, *m*, H-1).

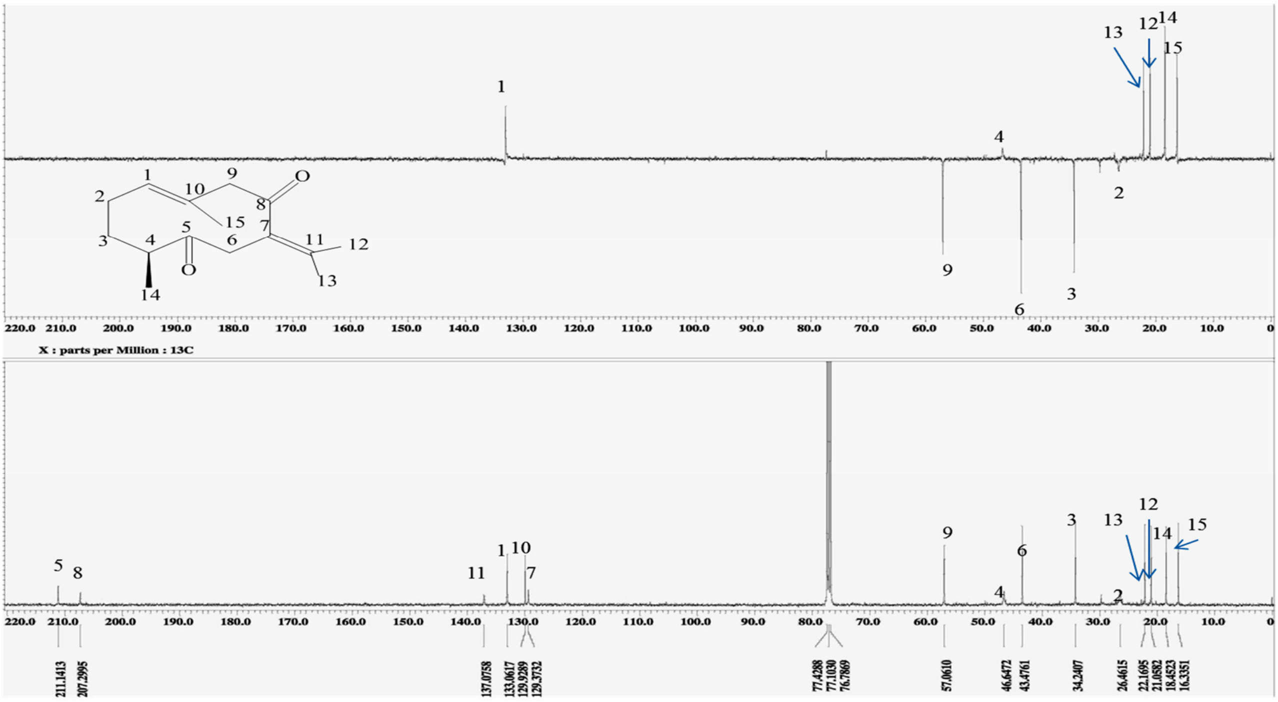

Figure S10. ^{13}C and DEPT spectra of Curdione (**5**) in $CDCl_3$. Curdione (**5**): ^{13}C NMR ($CDCl_3$, 100 MHz), δ: 16.6 (C-15), 18.6 (C-14), 21.2 (C-13), 19.9 (C-12), 30.1 (C-11), 129.9 (C-10), 55.9 (C-9), 214.2 (C-8), 53.7 (C-7), 44.3 (C-6), 211.0 (C-5), 46.8 (C-4), 34.1 (C-3), 26.5 (C-2), 131.5 (C-1).

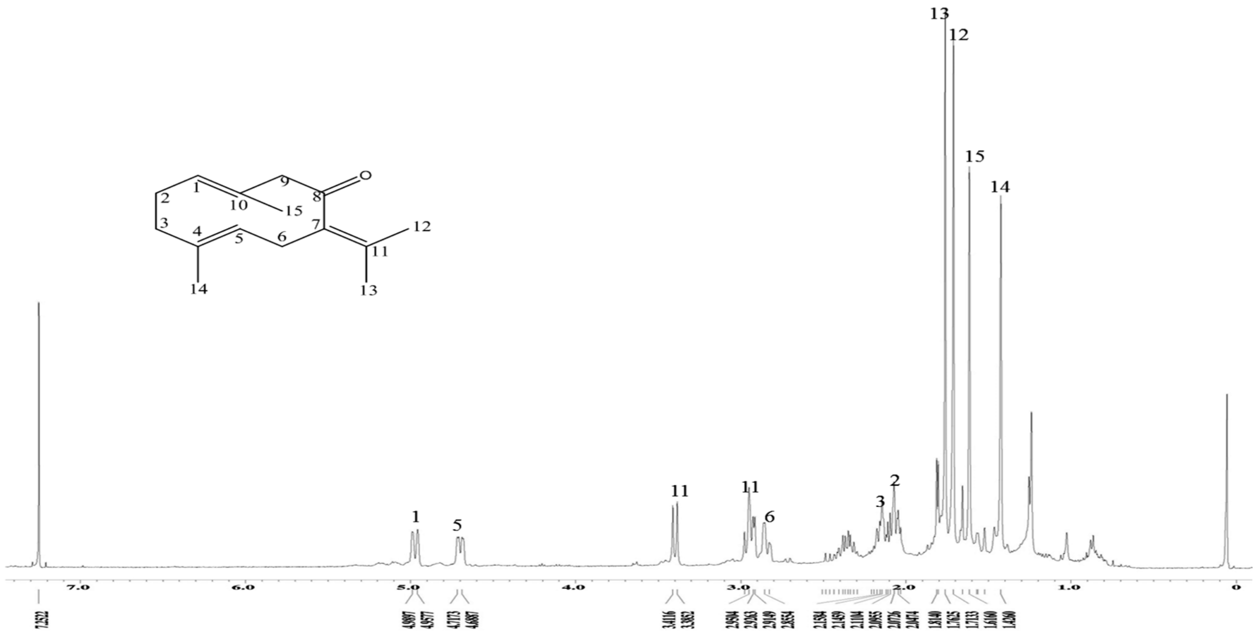

Figure S11. ^{1}H NMR spectrum of germacrone (**6**) in $CDCl_3$. Germacrone (**6**): ^{1}H NMR ($CDCl_3$, 400 MHz), δ: 1.62 (3H, *s*, H-15), 1.43 (3H, *s*, H-14), 1.76 (3H, *s*, H-13), 1.73 (1H, *s*, H-12), 3.42, 2.95 (2H, *dd*, *J* = 11, 3.68 Hz, H-9), 2.86 (2H, *m*, H-6), 4.71 (1H, *d*, *J* = 11 Hz, H-5), 2.15 (2H, *m*, H-3), 2.08, 2.35 (2H, *m*, H-2), 4.94 (1H, d, *J* = 11.8 Hz, H-1).

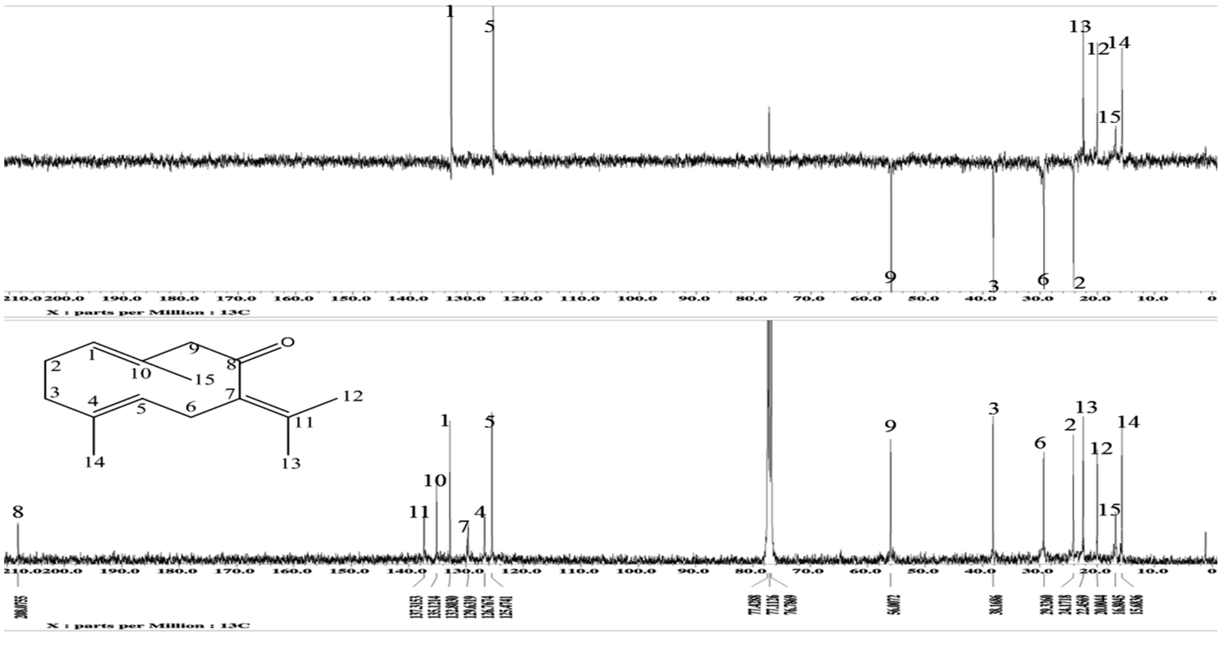

Figure S12. ^{13}C and DEPT spectra of Germacrone (**6**) in $CDCl_3$. Germacrone (**6**): ^{13}C NMR ($CDCl_3$, 100 MHz), δ: 16.8 (C-15), 15.6 (C-14), 22.4 (C-13), 20.0 (C-12), 137.0 (C-11), 135.1 (C-10), 56.0 (C-9), 208.0 (C-8), 129.0 (C-7), 29.3 (C-6), 125.4 (C-5), 126.0 (C-4), 38.1 (C-3), 24.0 (C-2), 132.8 (C-1).

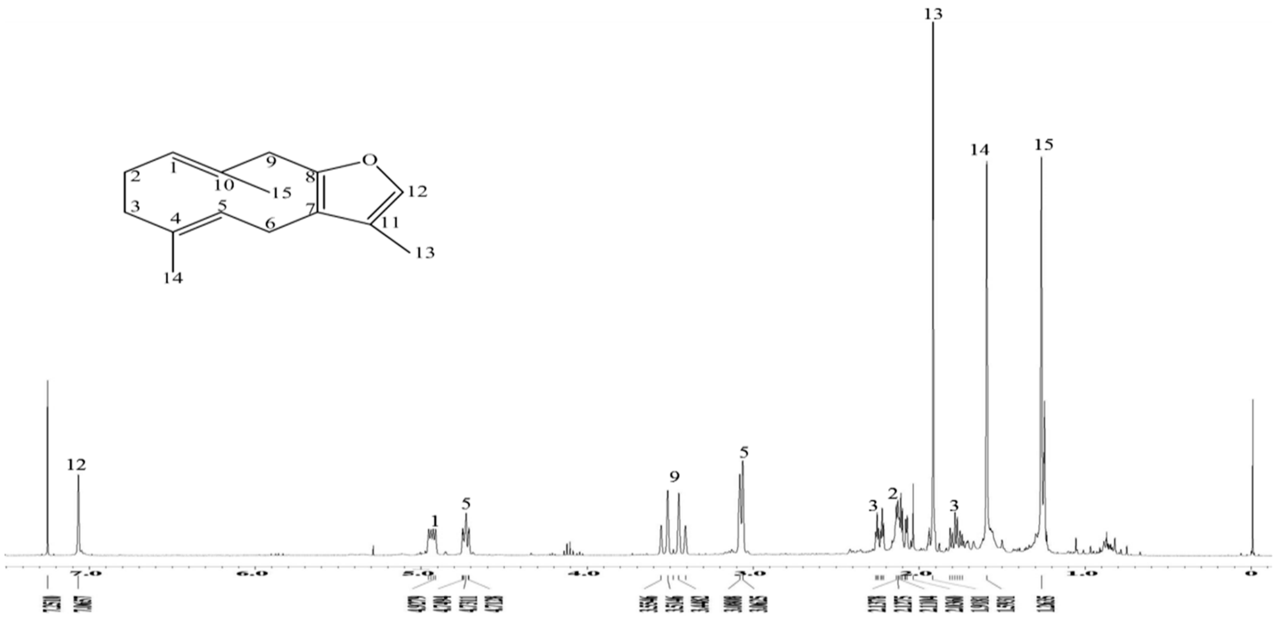

Figure S13. ^{1}H NMR spectrum of Furanodiene (**7**) in $CDCl_3$. Furanodiene (**7**): ^{1}H NMR ($CDCl_3$, 400 MHz), δ: 1.26 (3H, *s*, H-15), 1.59 (3H, *s*, H-14), 1.92 (3H, *s*, H-13), 5.98 (1H, *m*), 7.07 (1H, *s*, H-12), 3.51, 3.72 (2H-9, *d*, *J* = 16 Hz), 2.2, 1.59 (2H-6, *d*, *J* = 7 Hz), 4.73 (H-5, *t*, *J* = 7 Hz), 2.11 (2 H, m), 2.13 (2H, m) , 4.91 (H-1, m).

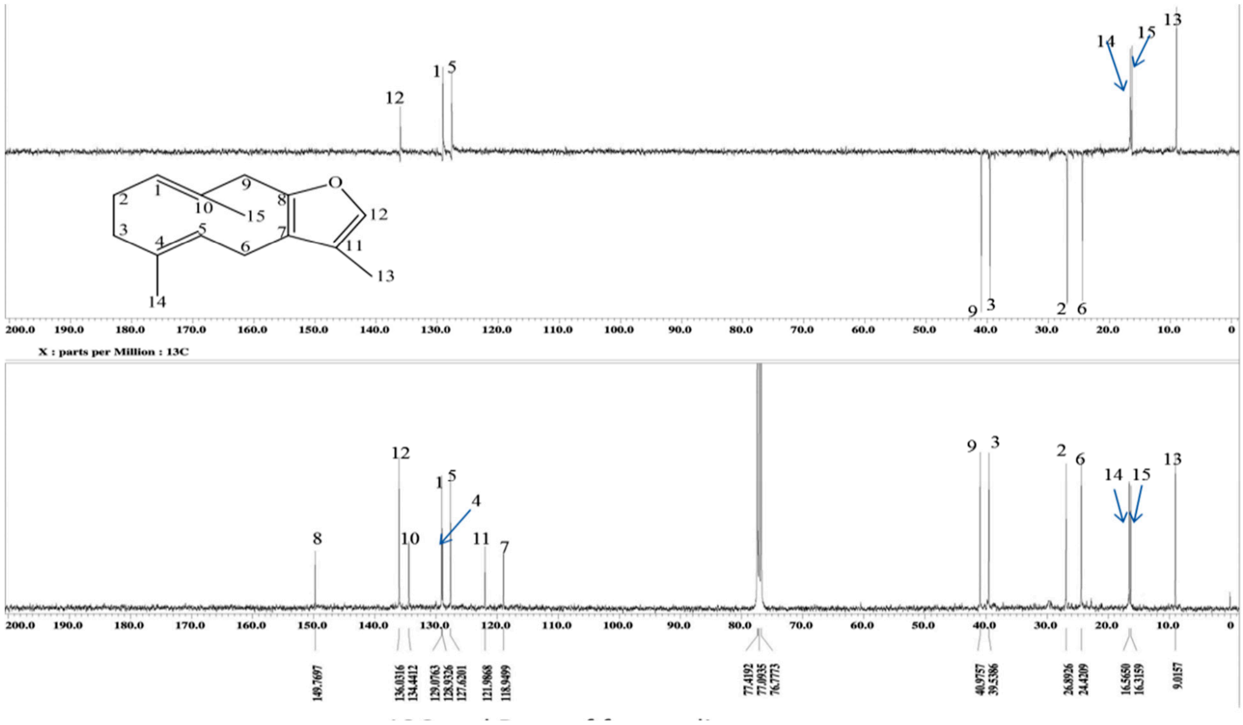

Figure S14. ^{13}C and DEPT spectra of Furanodiene (**7**) in $CDCl_3$. Furanodiene (**7**): ^{13}C NMR ($CDCl_3$, 100 MHz), δ: 16.3 (C-15), 16.7 (C-14), 9.0 (C-13), 136.0 (C-12), 121.9 (C-11), 134.4 (C-10), 40.9 (C-9), 140.8 (C-8), 118.9 (C-7), 24.4 (C-6), 127.6 (C-5), 128.9 (C-4), 39.5 (C-3), 26.9 (C-2), 129.0 (C-1).

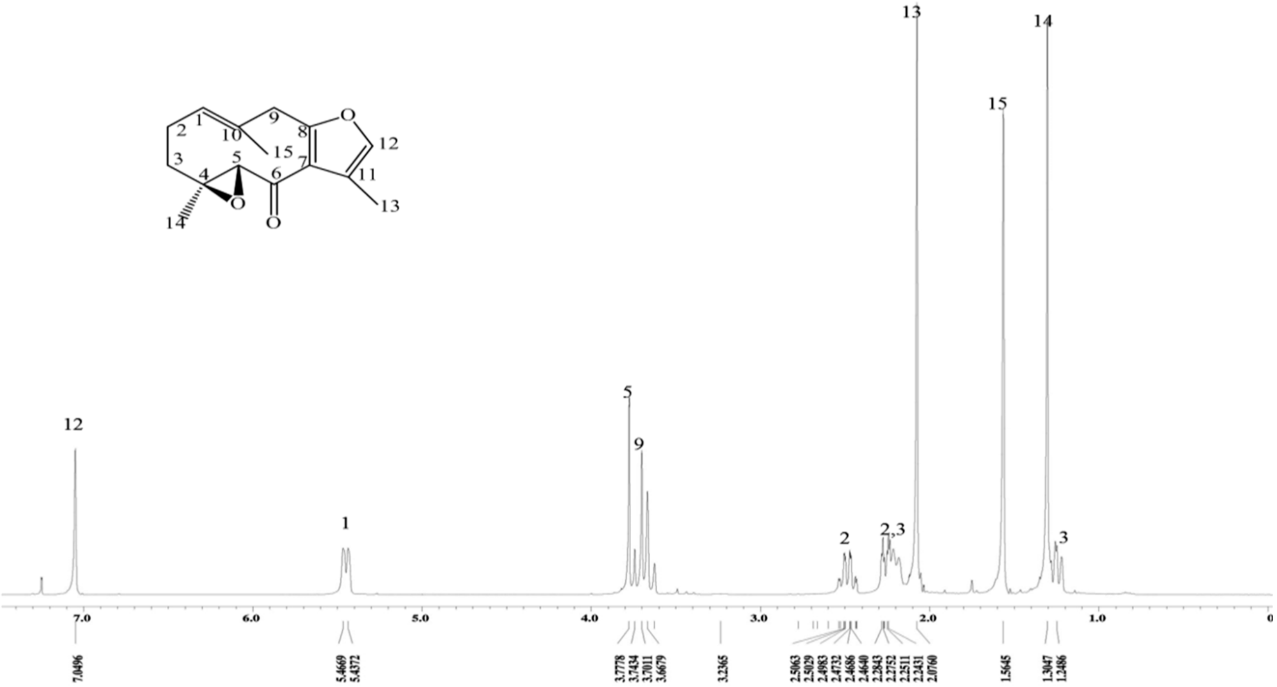

Figure S15. ^{1}H NMR spectrum of Zederone (**8**) in $CDCl_3$. Zederone (**8**): ^{1}H NMR ($CDCl_3$, 400 MHz), δ: 1.56 (3H, *br.s*, H-15), 1.30 (3H, *s*, H-14), 2.07 (3H, *s*, H-13), 7.04 (1H, *br.s*, H-12), 3.66, 3.70 (2H, *m*, H-9), 3.77 (1H, *s*, H-5), 1.24, 2.27 (2H, *m*, H-3), 2.24, 2.46 (2H, *m*, H-2), 5.46 (1H, *d*, *J* = 11.8 Hz, H-1).

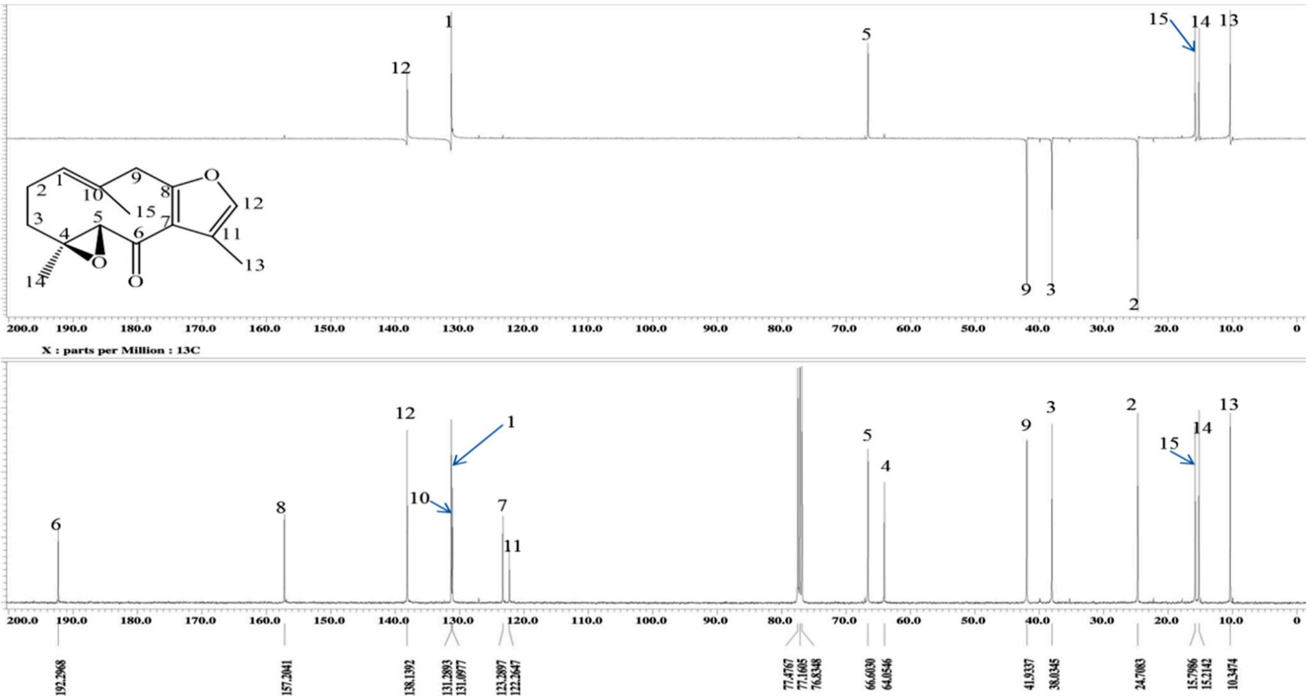

Figure S16. ^{13}C and DEPT spectra of Zederone (**8**) in $CDCl_3$. Zederone (**8**): ^{13}C NMR ($CDCl_3$, 100 MHz), δ: 15.8 (C-15), 15.2 (C-14), 10.3 (C-13), 138.1 (C-12), 122.2 (C-11), 131.1 (C-10), 41.9 (C-9), 157.2 (C-8), 123.2 (C-7), 192.2 (C-6), 66.6 (C-5), 64.0 (C-4), 38.0 (C-3), 24.7 (C-2), 131.2 (C-1).

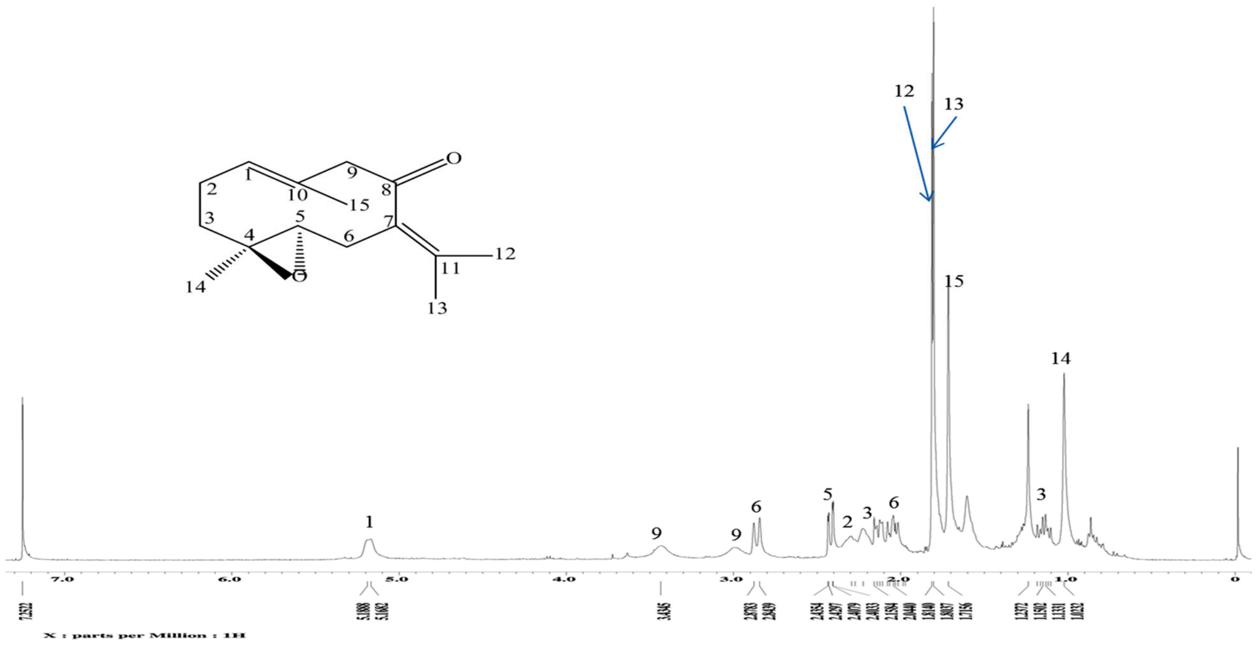

Figure S17. ^{1}H NMR spectrum of Germacrone-4,5-epoxide (**9**) in $CDCl_3$. Germacrone-4,5-epoxide (**9**): ^{1}H NMR ($CDCl_3$, 400 MHz), δ: 1.71 (3H, *s*, H-15), 1.02 (3H, *s*, H-14), 1.80 (3H, *s*, H-13), 1.81 (1H, *s*, H-12), 3.43 (2H, *br.s*, H-9), 2.88, 2.03 (2H, *br.d*, *J* = 15.12 Hz, H-6), 2.43 (1H, *d*, *J* = 1.84 Hz, H-3), 2.13 (2H, *m*, H-3), 2.24 (2H, *m*, H-2), 5.28 (1H, *br.d*, *J* = 9.15 Hz, H-1).

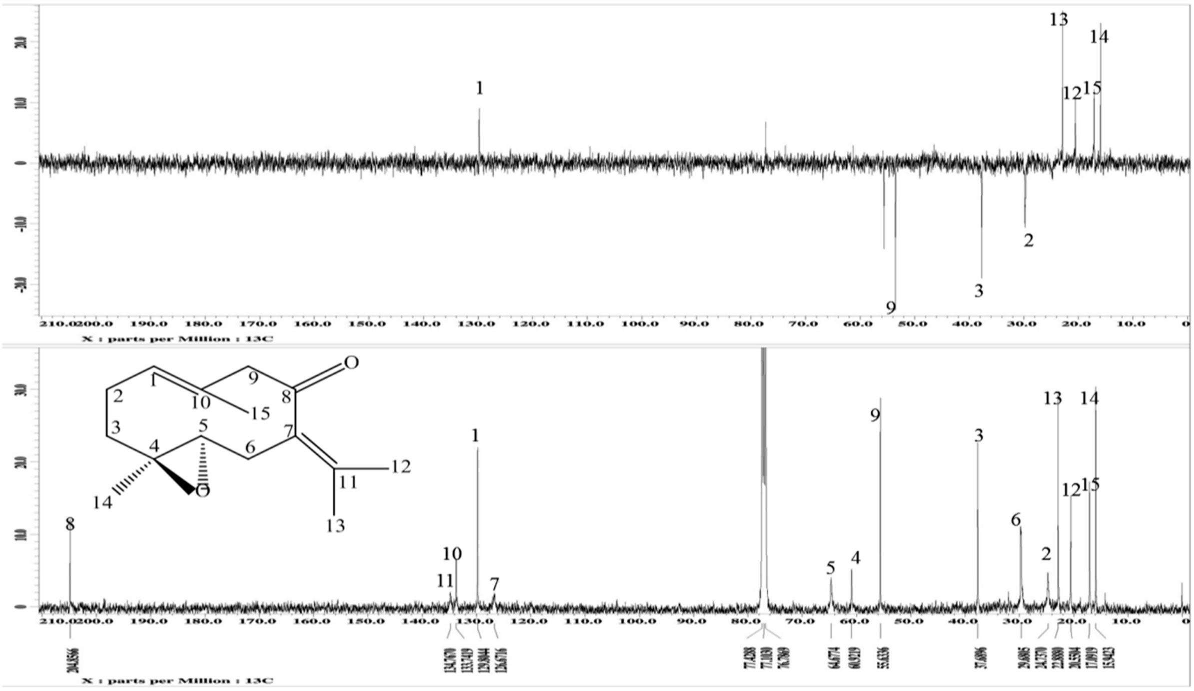

Figure S18. ^{13}C and DEPT spectra of Germacrone-4,5-epoxide (**9**) in $CDCl_3$. Germacrone-4,5-epoxide (**9**): ^{13}C NMR ($CDCl_3$, 100 MHz), δ: 17.0 (C-15), 15.9 (C-14), 22.8 (C-13), 20.5 (C-12), 133.9 (C-11), 133.7 (C-10), 55.6 (C-9), 204.8 (C-8), 126.7 (C-7), 29.7 (C-6), 64.3 (C-5), 60.9 (C-4), 37.7 (C-3), 24.7 (C-2), 129.8 (C-1).

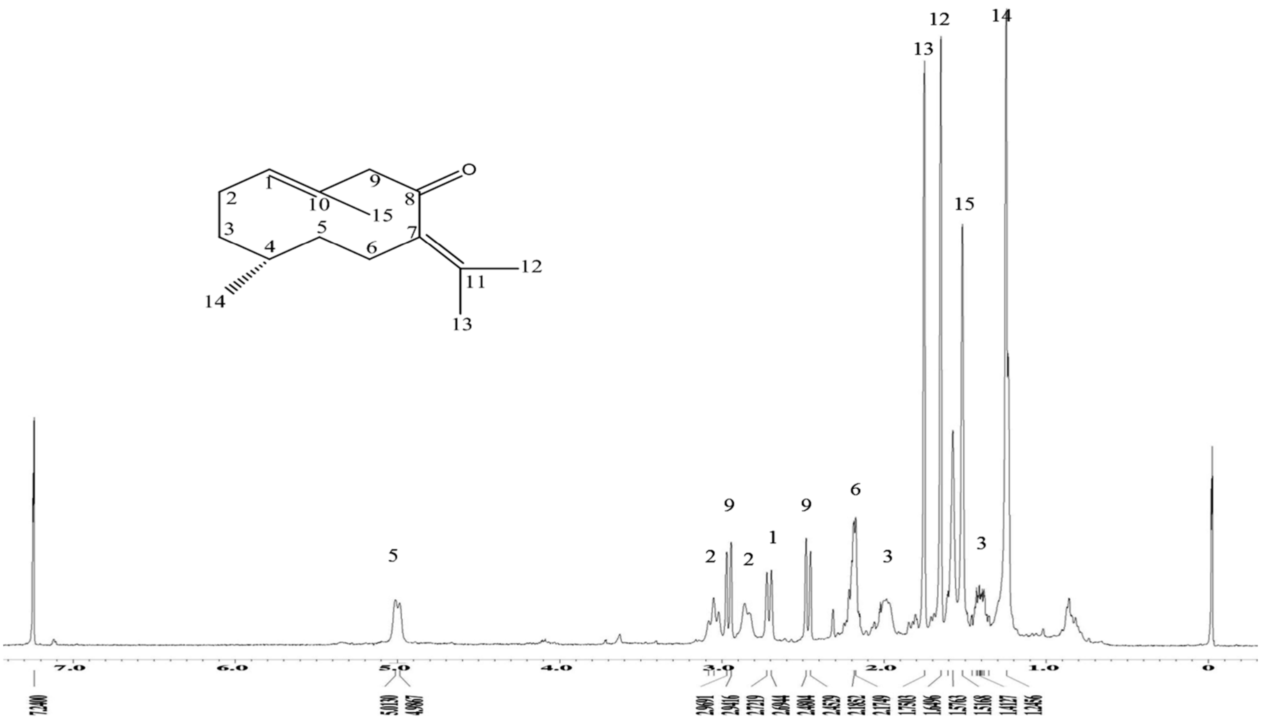

Figure S19. ^{1}H NMR spectrum of germacrone-1,10-epoxide (**9**) in $CDCl_3$. Germacrone-1,10-epoxide (**10**): ^{1}H NMR ($CDCl_3$, 400 MHz), δ: 1.51 (3H, *s*, H-15), 1.24 (3H, *s*, H-14), 1.75 (3H, *s*, H-13), 1.64 (3H, *s*, H-12), 2.46, 2.95 (2H, *d*, *J* = 11 Hz, H-9), 2.17 (2H, *d*, *J* = 4.12 Hz, H-6), 5.01 (1H, *m*, H-5), 1.40, 2.00 (2H, *m*, H-3), 2.85, 3.05 (1H, *d t*, *J* = 12.36 Hz, H-2), 2.70 (1H, *d*, *J* = 11 Hz, H-1).

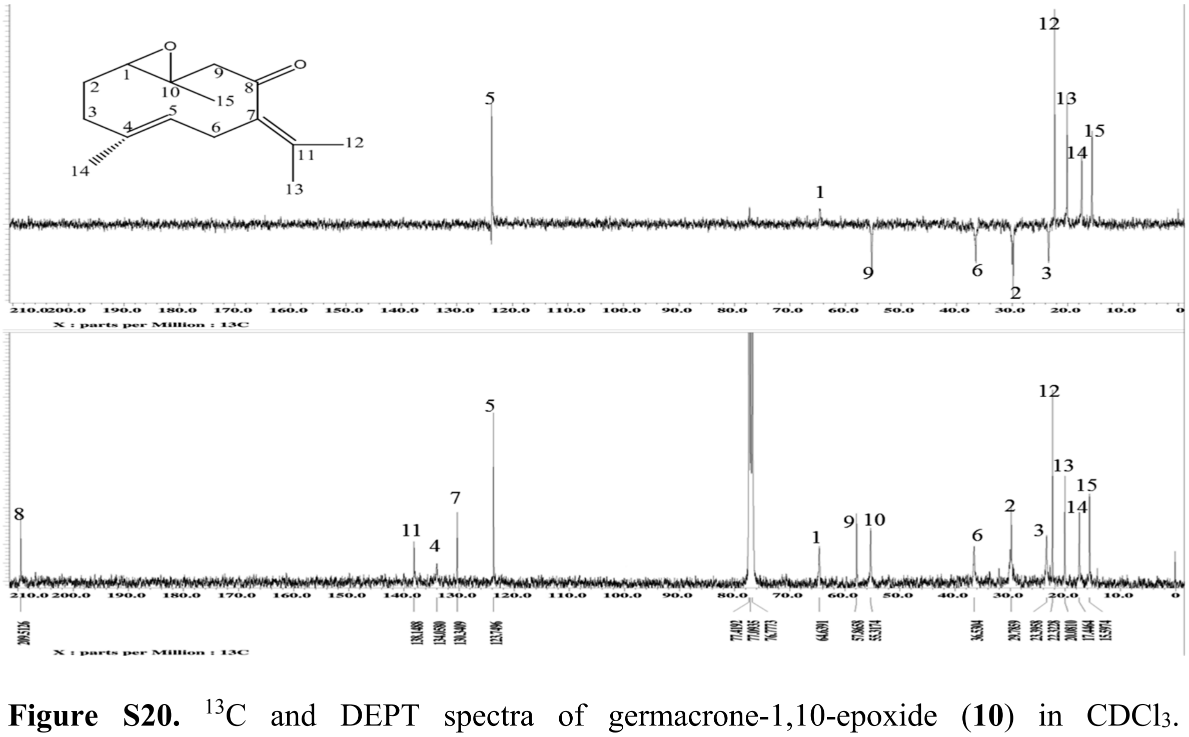

Figure S20. ^{13}C and DEPT spectra of germacrone-1,10-epoxide (**10**) in $CDCl_3$. Germacrone-1,10-epoxide (**10**): ^{13}C NMR ($CDCl_3$, 100 MHz), δ: 15.5 (C-15), 17.4 (C-14), 20.0 (C-13), 22.3 (C-12), 138.1 (C-11), 57.8 (C-10), 55.3 (C-9), 209.5 (C-8), 130.7 (C-7), 36.1 (C-6), 123.7 (C-5), 134.0 (C-4), 23.3 (C-3), 29.7 (C-2), 64.6 (C-1).

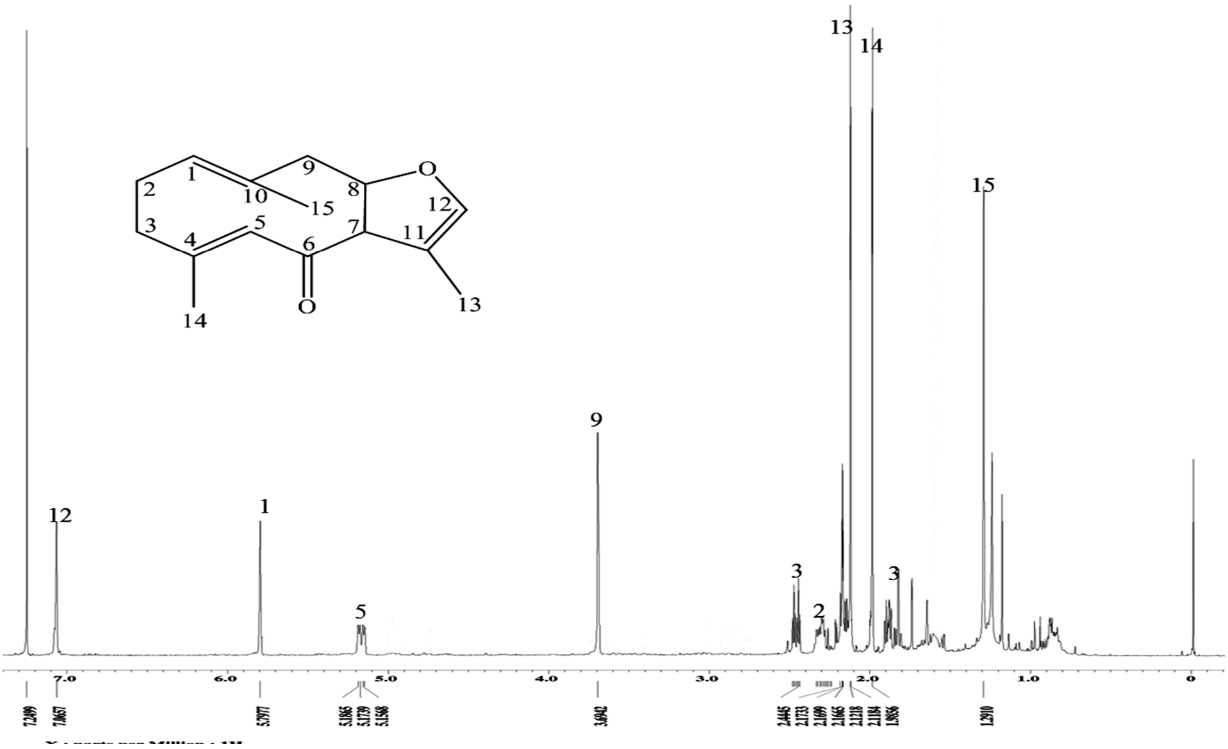

Figure S21. ^{1}H NMR spectrum of Furanodienone (**11**) in $CDCl_3$. Furanodienone (**11**): ^{1}H NMR ($CDCl_3$, 400 MHz), δ: 1.29 (3H, *s*, H-15), 1.98 (3H, *s*, H-14), 2.12 (3H, *s*, H-13), 7.06 (1H, *br.s*, H-12), 3.73 (2H, *br.s*, H-9), 5.80 (1H, *br.s*, H-5), 1.90 (2H, *dt*, H-3), 2.17 (2H, *dt*, H-2), 5.15 (1H, *dd*, *J* = 4.12, 10.9 Hz, H-1).

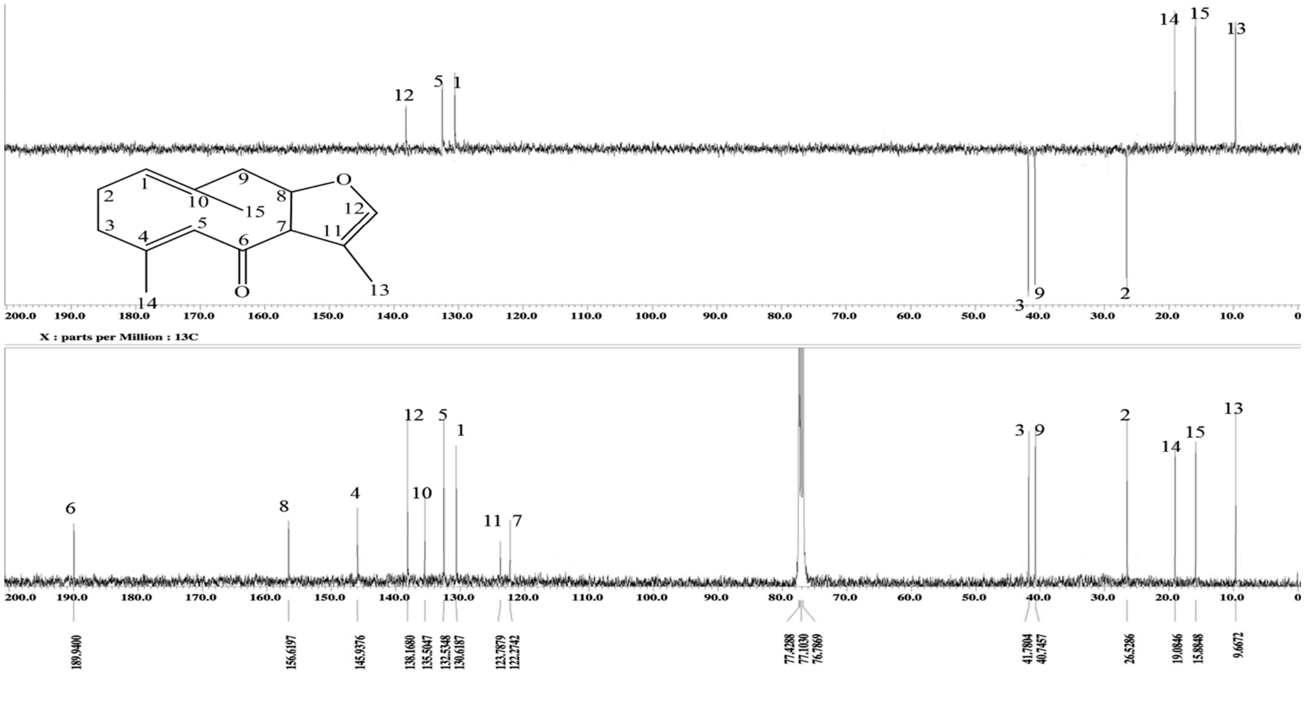

Figure S22. ^{13}C and DEPT spectra of Furanodienone (**11**) in $CDCl_3$. Furanodienone (**11**): ^{13}C NMR ($CDCl_3$, 100 MHz), δ: 15.8 (C-15), 19.0 (C-14), 9.6 (C-13), 138.1 (C-12), 123.7 (C-11), 135.5(C-10), 40.7 (C-9), 156.6 (C-8), 122.2 (C-7), 189.9 (C-6), 132.5 (C-5), 145.9 (C-4), 41.7 (C-3), 26.5 (C-2), 130.6 (C-1).

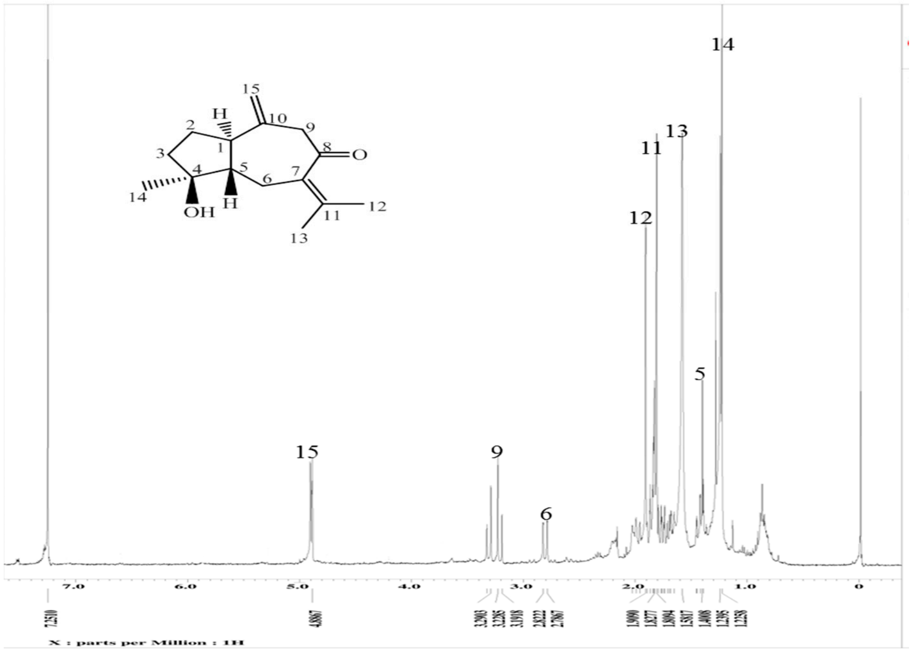

Figure S23. ^{1}H NMR spectrum of Isoprocurcumenol (**12**) in $CDCl_3$. Isoprocurcumenol (**12**): ^{1}H NMR ($CDCl_3$, 400 MHz), δ: 4.90 (2H, *d*, *J* = 6.88 Hz, H-15), 1.24 (3H, *s*, H-14), 1.82 (3H, *s*, H-13), 1.92 (3H, *s*, H-12), 2.16 (2H, *s*, H-9), 2.81 (2H, *d*, *J* = 14.2 Hz, H-6), 1.40 (1H, *m*, H-5), 1.39 (2H, *m*, H-3), 1.21 (2H, *m*, H-2), 3.22 (1H, *q*, *J* = 14.68 Hz, H-1).

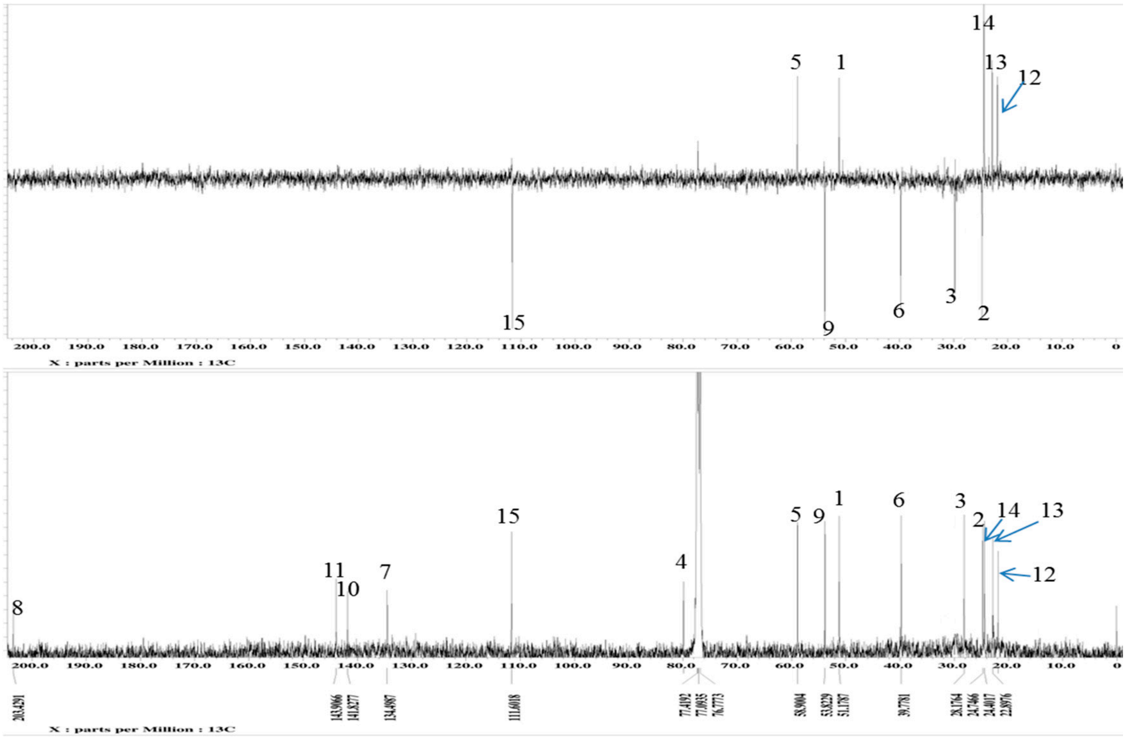

Figure S24. ^{13}C and DEPT spectra of isoprocurcumenol (**12**) in $CDCl_3$. Isoprocurcumenol (**12**): ^{13}C NMR ($CDCl_3$, 100 MHz), δ: 111.6 (C-15), 24.4 (C-14), 22.8 (C-13), 21.9 (C-12), 143.9 (C-11), 141.3 (C-10), 53.8 (C-9), 203 (C-8), 134.5 (C-7), 39.8 (C-6), 58.9 (C-5), 77.4 (C-4), 28.2 (C-3), 24.7 (C-2), 51.2 (C-1).

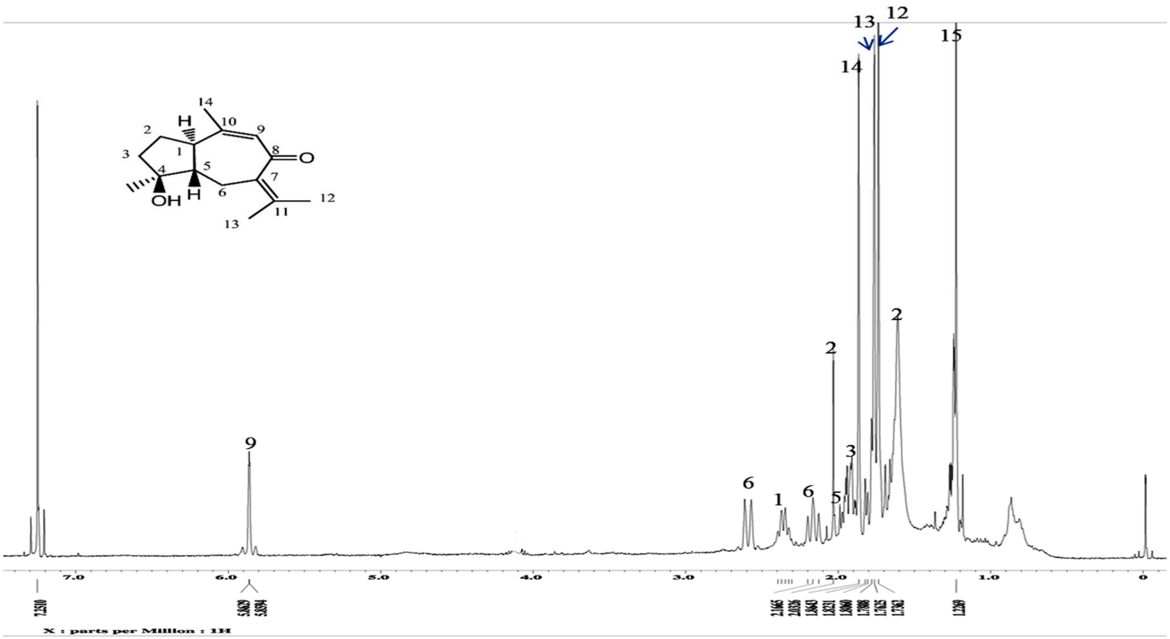

Figure S25. ^{1}H NMR spectrum of procurcumenol (**13**) in $CDCl_3$. Procurcumenol (**13**): ^{1}H NMR ($CDCl_3$, 400 MHz), δ: 1.22 (3H, *s*, H-15), 1.86 (3H, *s*, H-14), 1.76 (3H, *s*, H-13), 1.73 (3H, *s*, H-12), 5.85 (1H, *d*, J = 1.4 Hz, H-9), 2.16 (1H, *dd*, J = 13.2, 15.6 Hz, H_a), 2.57 (1H, d, J = 15.6 Hz, H_b-6), 1.91 (1H, *m*, H-5), 1.88 (2H, *m*, H-3), 1.66, 1.94 (2H, *m*, H-2), 2.34 (2H, *dd*, J = 8.7, 9.6 Hz, H-1).

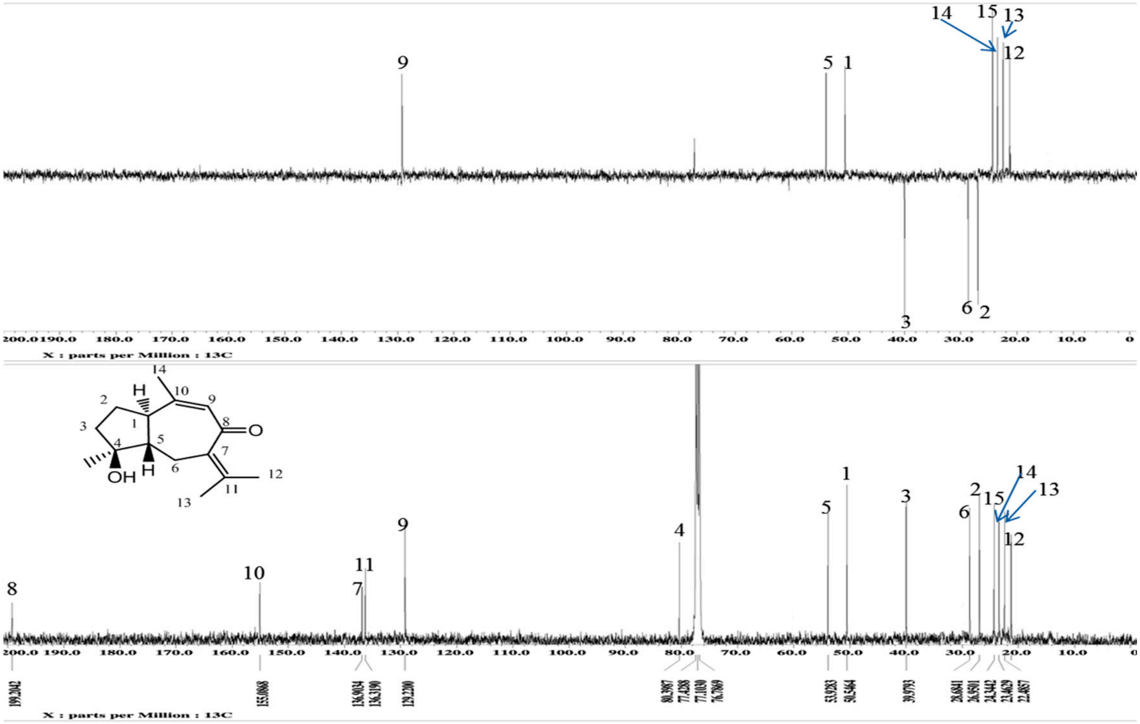

Figure S26. ^{13}C and DEPT spectra of procurcumenol (**13**) in $CDCl_3$. Procurcumenol (**13**): ^{13}C NMR ($CDCl_3$, 100 MHz), δ: 24.3 (C-15), 23.4 (C-14), 22.4 (C-13), 21.3 (C-12), 136.3 (C-11), 155.1 (C-10), 129.2 (C-9), 199.2 (C-8), 136.9 (C-7), 28.6 (C-6), 53.9 (C-5), 80.3 (C-4), 39.9 (C-3), 26.9 (C-2), 50.5 (C-1).

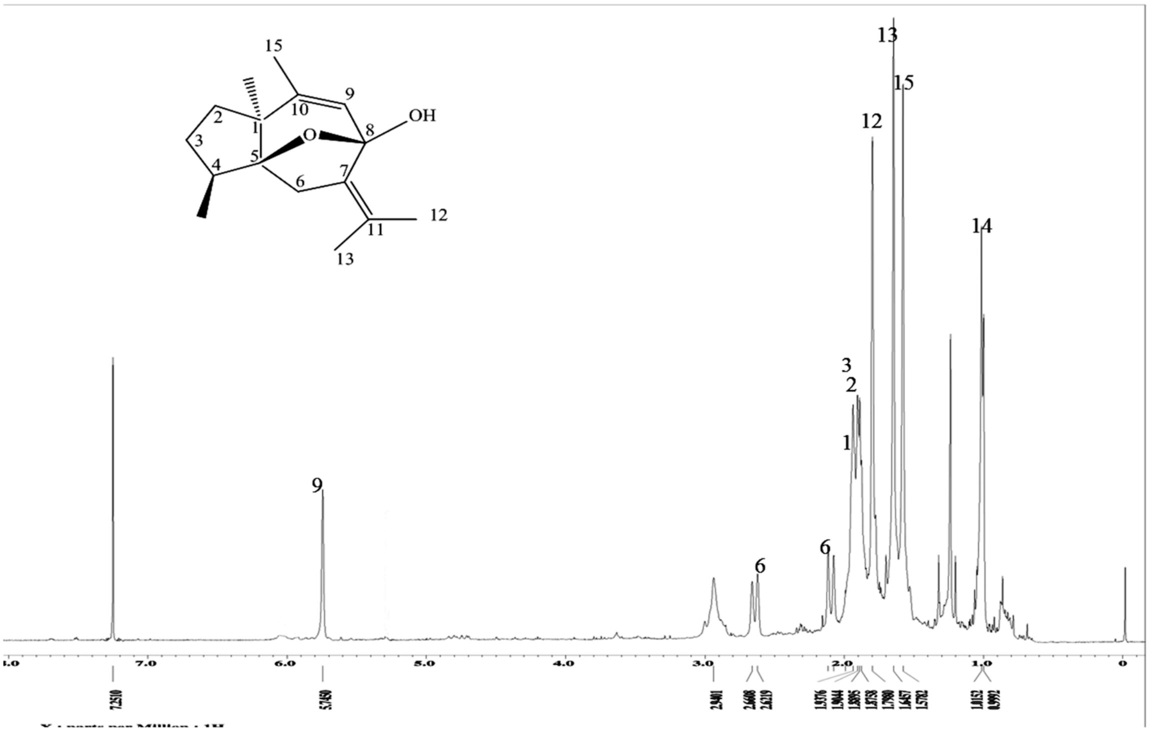

Figure S27. ^{1}H NMR spectrum of Curcumenol (**14**) in $CDCl_3$. Curcumenol (**14**): ^{1}H NMR ($CDCl_3$, 400 MHz), δ: 1.79 (3H, *s*, H-15), 1.01 (3H, *d*, *J* = 6.4 Hz, H-14), 1.61 (3H, *s*, H-13), 1.54 (3H, *s*, H-12), 5.74 (1H, *br.s*, H-9), 2.11, 2.66 (2H, *d*, *J* = 15.4 Hz, H-6), 1.9 (6H, *m*, H-2, 3, 4), 2.07 (1H. *d*. *J* = 15.6 Hz, H-1).

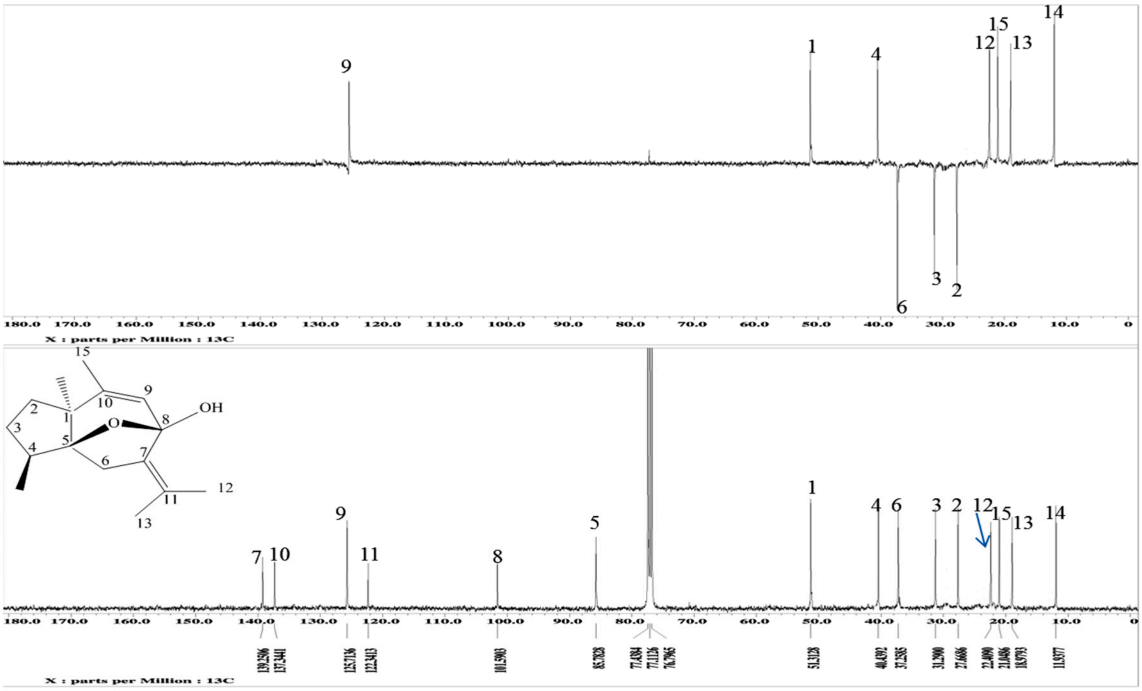

Figure S28. ^{13}C and DEPT spectra of curcumenol (**14**) in $CDCl_3$. Curcumenol (**14**): ^{13}C NMR ($CDCl_3$, 100 MHz), δ: 21.0 (C-15), 11.9 (C-14), 18.9 (C-13), 22.4 (C-12), 122.3 (C-11), 137.2 (C-10), 125.7 (C-9), 101.6 (C-8), 139.2 (C-7), 37.2 (C-6), 85.8 (C-5), 40.4 (C-4), 31.2 (C-3), 27.6 (C-2), 51.3 (C-1).

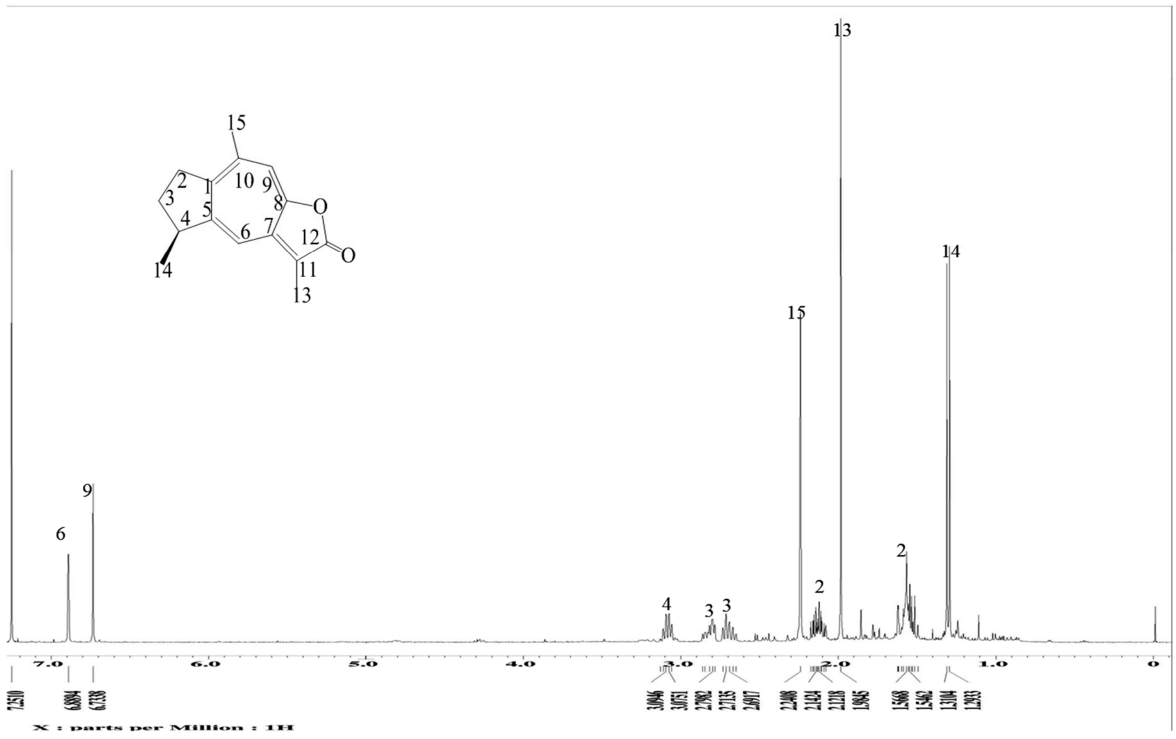

Figure S29. ^{1}H NMR spectrum of Gweicurculactone (**15**) in $CDCl_3$. Gweicurculactone (**15**): ^{1}H NMR ($CDCl_3$, 400 MHz), δ: 2.24 (3H, *s*, H-15), 1.30 (3H, *d*, *J* = 6.84 Hz, H-14), 1.98 (3H, *s*, H-13), 6.73 (1H, *s*, H-9), 6.88 (1H, *s*, H-6), 3.08 (1H, *q*, *J* = 14.2 Hz, H-4), 2.81, 2.67 (2H, *m*, H-3), 1.53 (1H, *m*, H-2), 2.12 (1H, *m*, H-1).

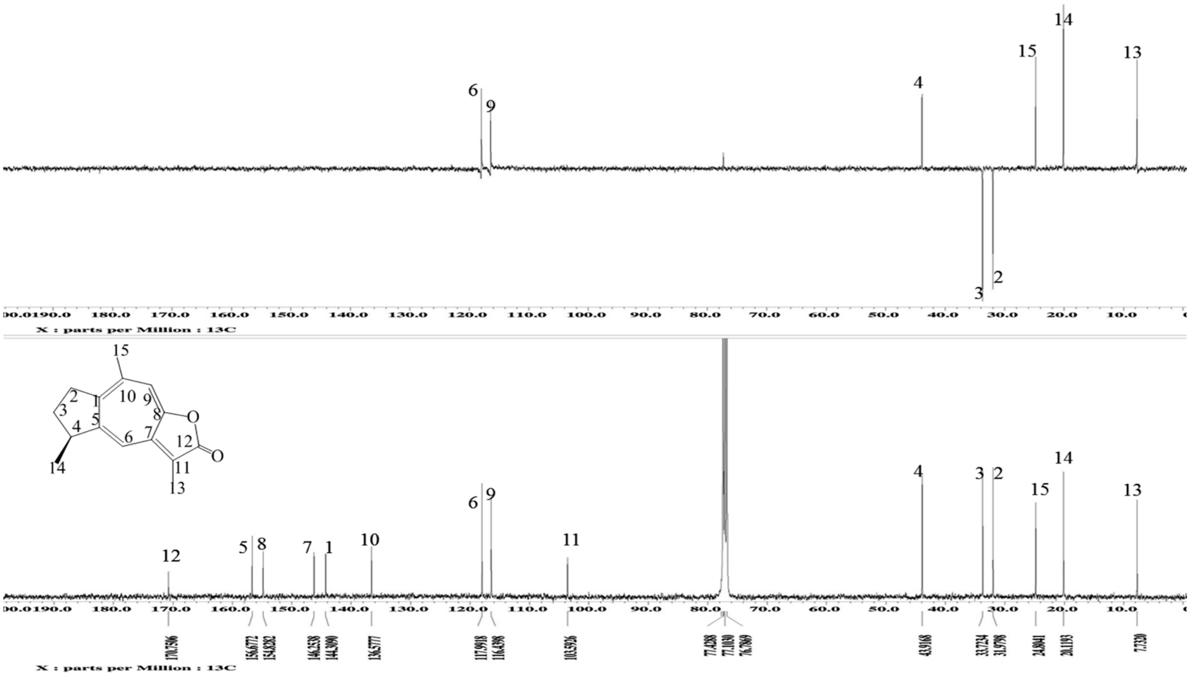

Figure S30. ^{13}C and DEPT spectra of Gweicurculactone (**15**) in $CDCl_3$. Gweicurculactone (**15**): ^{13}C NMR ($CDCl_3$, 100 MHz), δ: 24.8 (C-15), 20.1 (C-14), 7.7 (C-13), 170.7 (C-12), 103 (C-11), 136.5 (C-10), 116.4 (C-9), 154.8 (C-8), 146.2 (C-7), 117.9 (C-6), 156.6 (C-5), 43.9 (C-4), 33.3 (C-3), 31.9 (C-2), 144.2 (C-1).

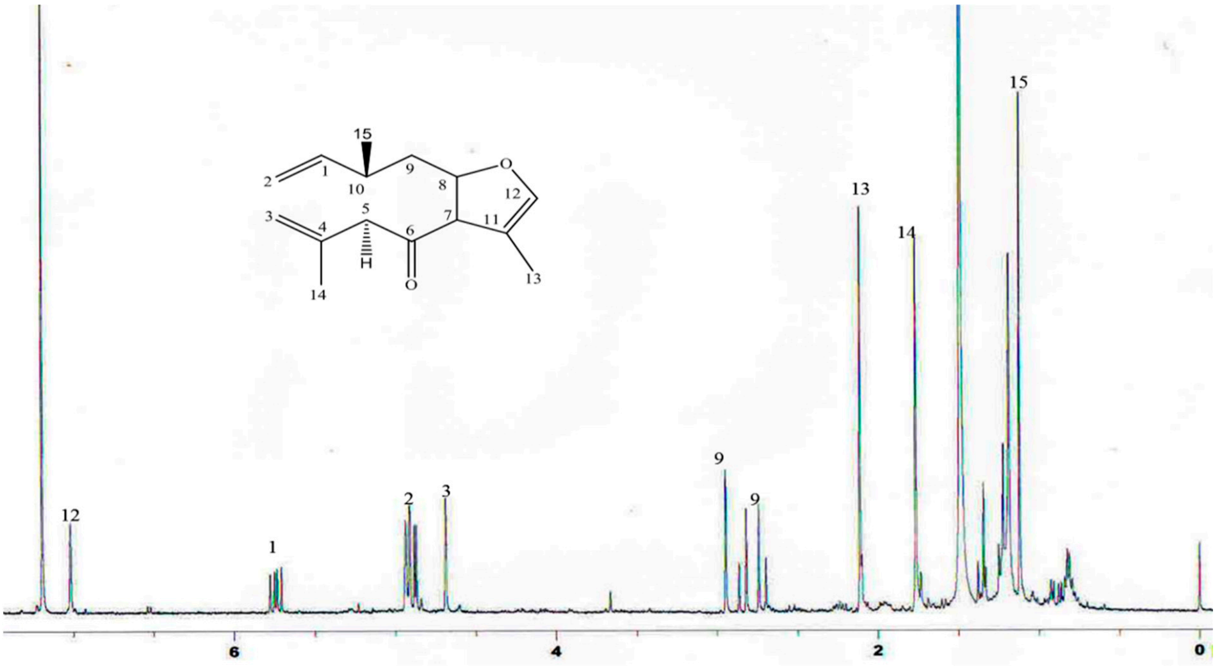

Figure S31. ^{1}H NMR spectrum of curcuzenone (**16**) in $CDCl_3$. Curcuzenone (**14**): ^{1}H NMR ($CDCl_3$, 400 MHz), δ: 1.18 (3H, *s*, H-15), 1.76 (3H, *s*, H-14), 2.20 (3H, *s*, H-13), 7.01 (1H, *s*, H-12), 2.94, 2.74 (2H, *d*, *J* = 17.2 Hz, H-9), 2.94 (1H, *s*, H-5), 4.84 (2H, *s*, H-3), 4.93 (2H, *s*, H-2), 5.77 (1H, *dd*, *J* = 10.8, 17 Hz, H-1).

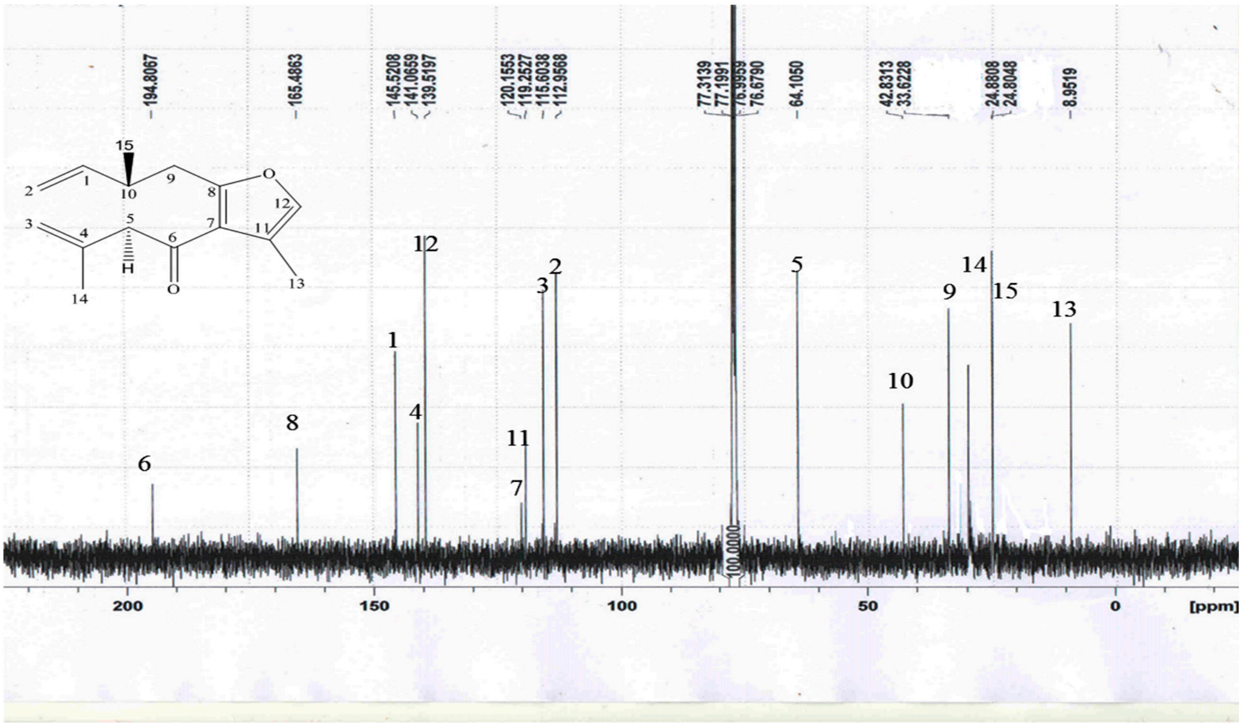

Figure S32. ^{13}C and DEPT spectra of Curcuzenone (**16**) in $CDCl_3$. Curcuzenone (**14**): ^{13}C NMR ($CDCl_3$, 100 MHz), δ: 24.8 (C-15), 24.9 (C-14), 8.9 (C-13), 139.5 (C-12), 119.2 (C-11), 42.8 (C-10), 33.6 (C-9), 165.4 (C-8), 120.1 (C-7), 194.8 (C-6), 64.1 (C-5), 141.0 (C-4), 115.6 (C-3), 112.9 (C-2), 145.5 (C-1).

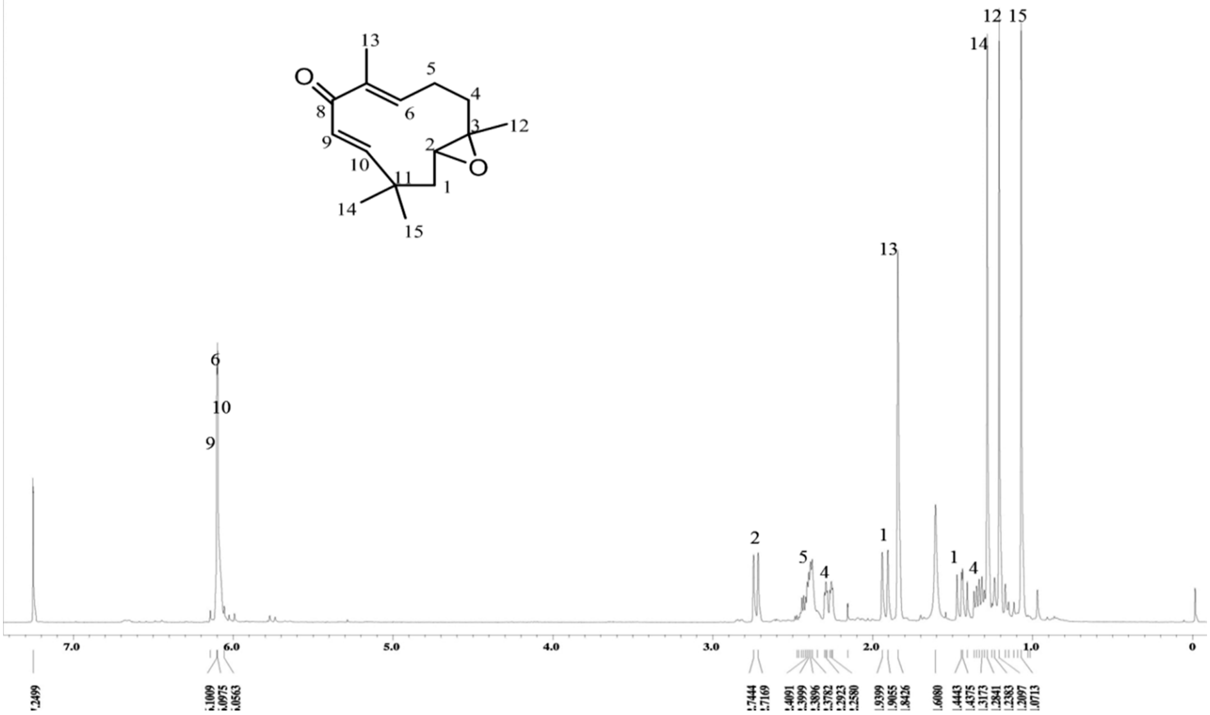

Figure S33. ^{1}H NMR spectrum of zerumbone epoxide (**17**) in $CDCl_3$. Zerumbone epoxide (**17**): ^{1}H NMR ($CDCl_3$, 400 MHz), δ: 1.07 (3H, *s*, H-15), 1.28 (3H, *s*, H-14), 1.84 (3H, *s*, H-13), 1.20 (3H, *s*, H-12), 6.10 (2H, *d*, *J* = 1.36 Hz, H-9), 6.1 (1H, *d*, *J* = 12.3 Hz, H-6), 1.94 (1H, *d*, *J* = 13 Hz, H-5), 1.30–1.38, 2.18–2.26 (2H, *m*, H-4), 2.74 (1H, *d*, *J* = 11 Hz, H-2), 1.44 (2H, *dd*, *J* = 2.7, 11.4 Hz).

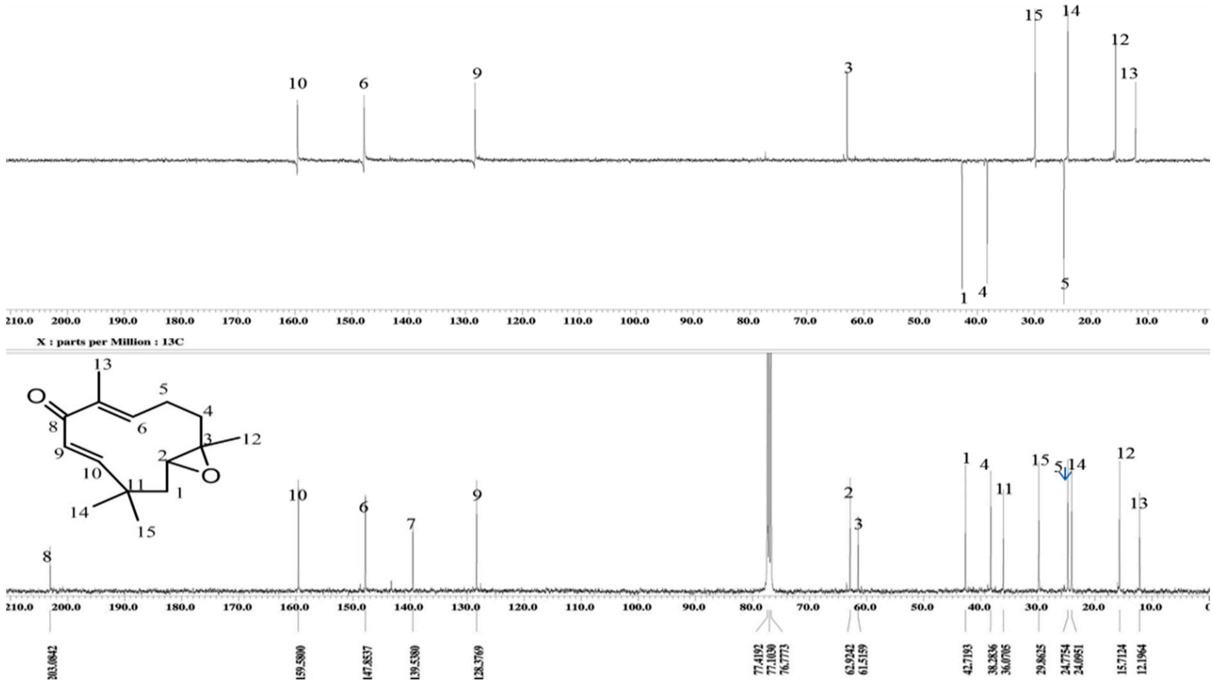

Figure S33. ^{13}C and DEPT spectra of Zerumbone epoxide (**17**) in $CDCl_3$. Zerumbone epoxide (**17**): ^{13}C NMR ($CDCl_3$, 100 MHz), δ: 29.8 (C-15), 24.0 (C-14), 12.1 (C-13), 15.7 (C-12), 36.0 (C-11), 159.5 (C-10), 128.3 (C-9), 203.0 (C-8), 139.5 (C-7), 147.8 (C-6), 24.7 (C-5), 38.2 (C-4), 61.9 (C-3), 62.5 (C-2), 42.7 (C-1).

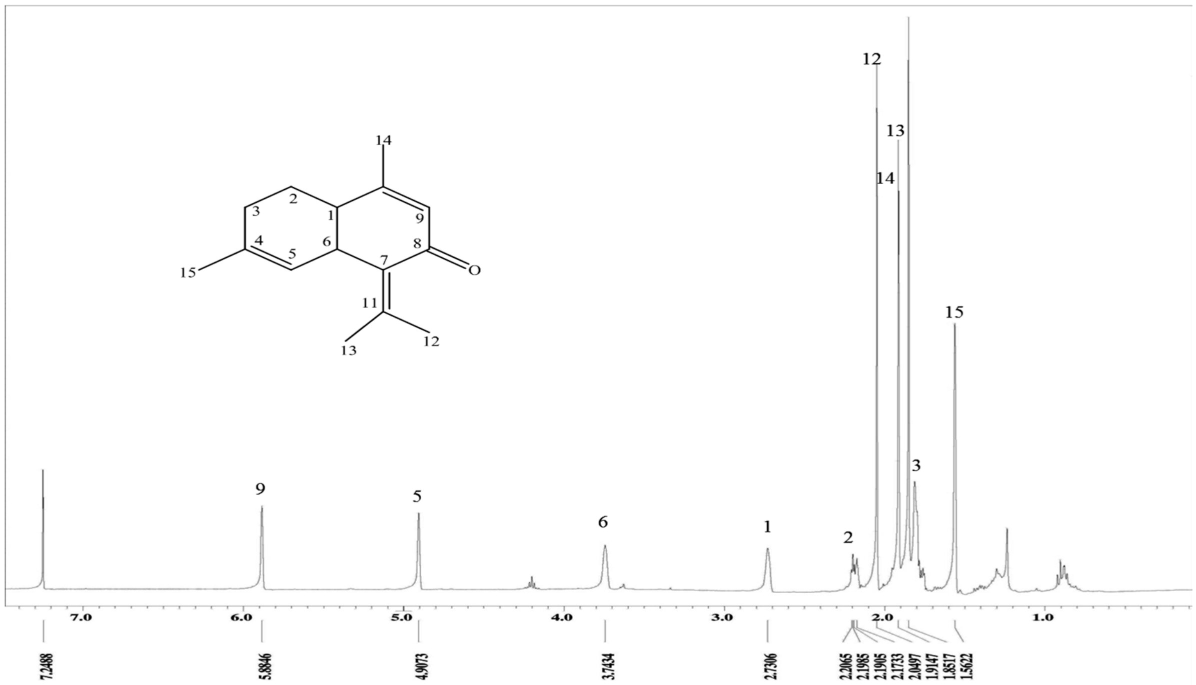

Figure S35. ^{1}H NMR spectrum of comosone II (**18**) in $CDCl_3$. Comosone II (**18**): ^{1}H NMR ($CDCl_3$, 400 MHz), δ: 1.56 (3H, *s*, H-15), 1.91 (3H, *s*, H-14), 1.85 (3H, *s*, H-13), 2.04 (1H, *br.s*, H-12), 5.88 (1H, *s*, H-9), 3.74 (1H, *br.s*, H-6), 4.90 (1H, *br.s*, H-5), 1.81 (2H, *m*, H-3), 1.82, 2.17 (2H, *m*, H-2). 2.73 (1H, *m*, H-1).

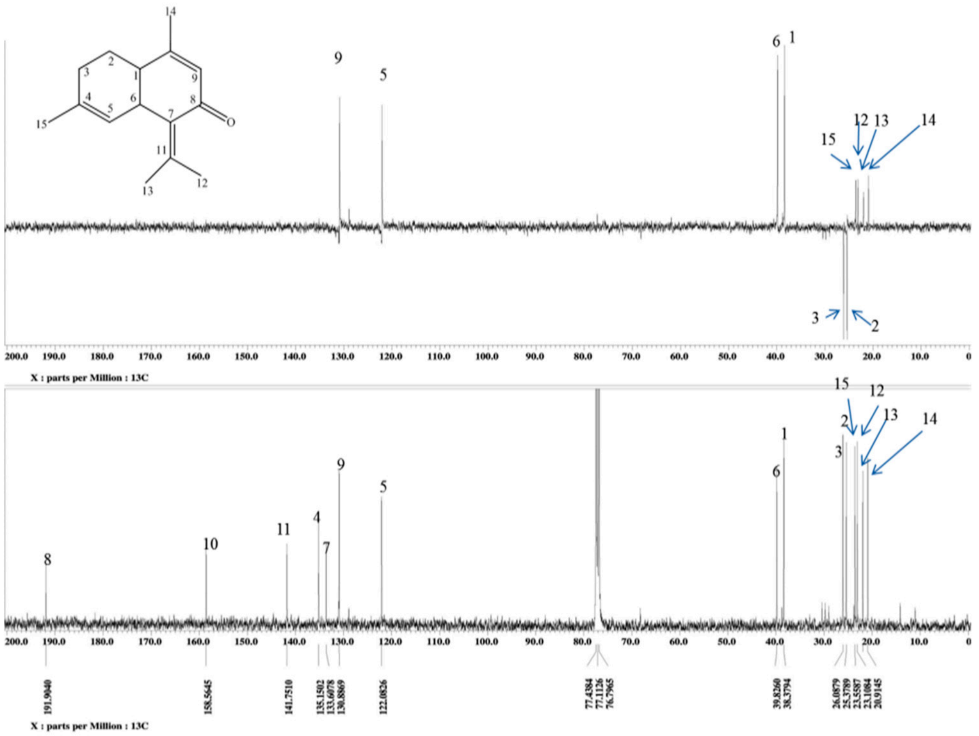

Figure S36. ^{13}C and DEPT spectra of Comosone II (**18**) in $CDCl_3$. Comosone II (**18**): ^{13}C NMR ($CDCl_3$, 100 MHz), δ: 23.5 (C-15), 20.9 (C-14), 21.9 (C-13), 23.1 (C-12), 141.8 (C-11), 158.6 (C-10), 130.8 (C-9), 191.9 (C-8), 133.6 (C-7), 39.8 (C-6), 122.1 (C-5), 135.1 (C-4), 26.0 (C-3), 25.4 (C-2), 38.3 (C-1).

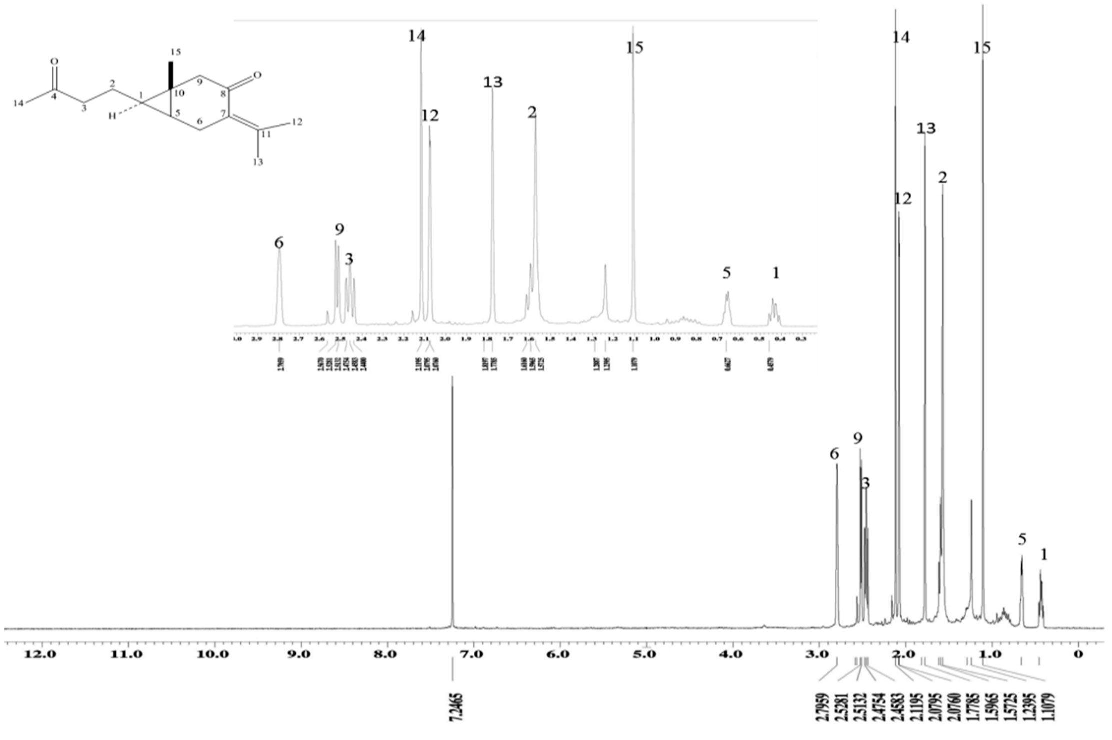

Figure S37. ^{1}H NMR spectrum of curcumenone (**19**) in $CDCl_3$. Curcumenone (**19**): ^{1}H NMR ($CDCl_3$, 400 MHz), δ: 1.10 (3H, *s*, H-15), 2.12 (3H, *s*, H-14), 1.77 (3H, *s*, H-13), 2.07 (3H, *s*, H-12), 2.52 (2H, *d*, *J* = 15.6 Hz, H-9), 2.8 (2H, *m*, H-6), 0.67 (1H, *q*, *J* = 4.56 Hz, H-5), 2.47 (2H, *t*, *J* = 7.36 Hz, H-3), 1.64 (2H, *q*, *J* = 7.32 Hz, H-2), 0.43 (1H, *dt*, *J* = 4.56 Hz, 7.32 Hz, H-1).

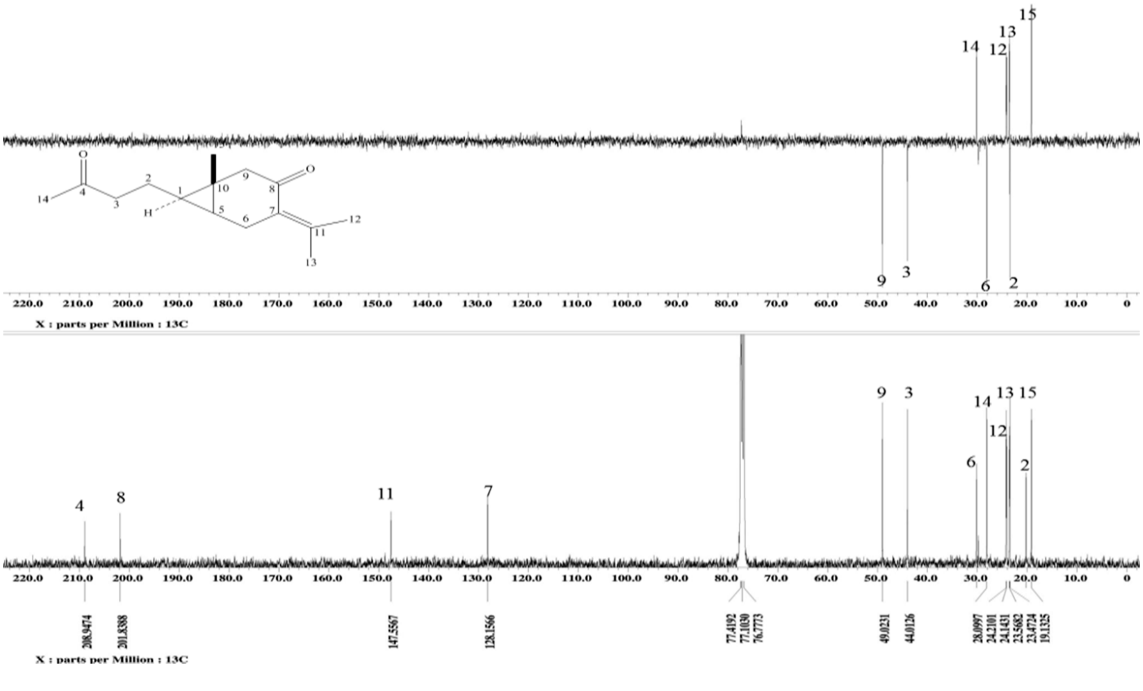

Figure S38. ^{13}C and DEPT spectra of curcumenone (**19**) in $CDCl_3$. Curcumenone (**19**): ^{13}C NMR ($CDCl_3$, 100 MHz), δ: 19.1 (C-15), 30.1 (C-14), 23.5 (C-13), 23.5 (C-12), 147.6 (C-11), 20.2 (C-10), 49.0 (C-9), 201.9 (C-8), 128.1 (C-7), 28.0 (C-6), 24.2 (C-5), 209.0 (C-4), 44.0 (C-3), 23.4 (C-2), 24.1 (C-1).

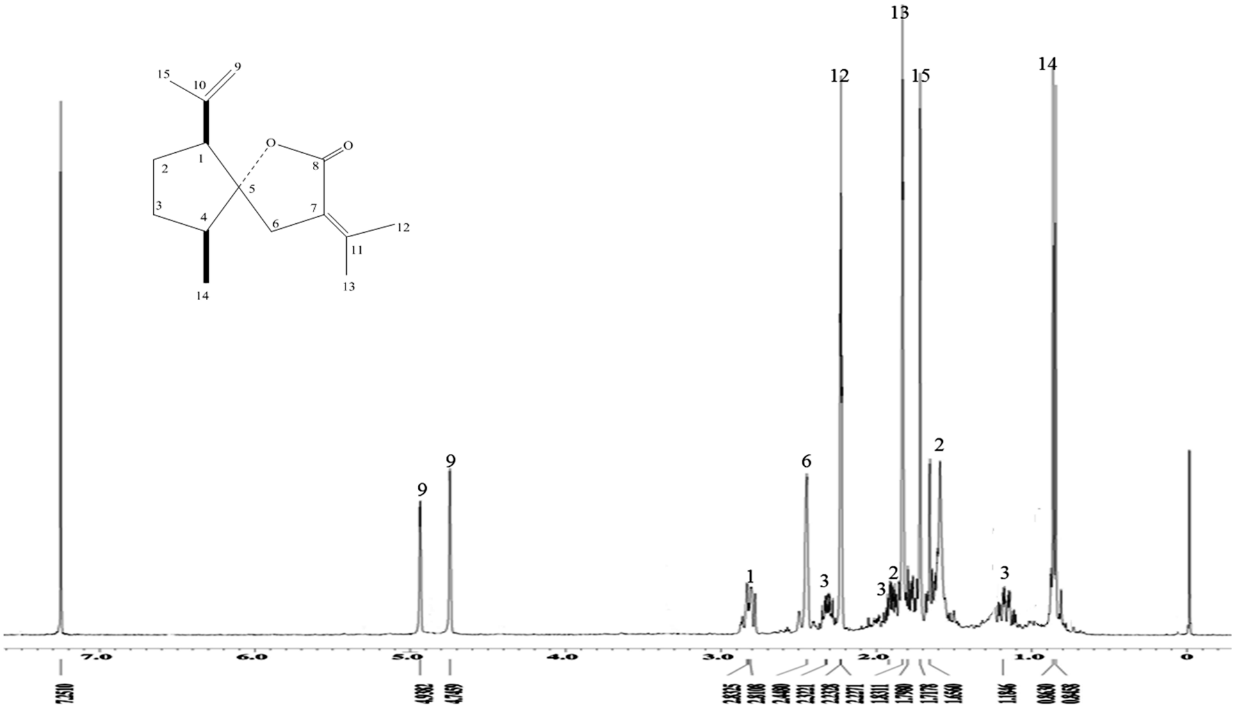

Figure S39. ^{1}H NMR spectrum of Curcumanolide A (**20**) in $CDCl_3$. Curcumanolide A (**20**): ^{1}H NMR ($CDCl_3$, 400 MHz), δ: 1.82 (3H, *s*, H-15), 0.86 (3H, *s*, H-14), 1.83 (3H, *s*, H-13), 2.22 (3H, *br.s*, H-12), 4.74, 4.93 (2H, *s*, H-9), 2.44 (2H, *br.s*, H-6), 1.83, 2.22 (2H, *m*, H-3), 1.65, 1.82 (2H, *m*, H-2), 2.83 (2 1H, *m*, H-1).

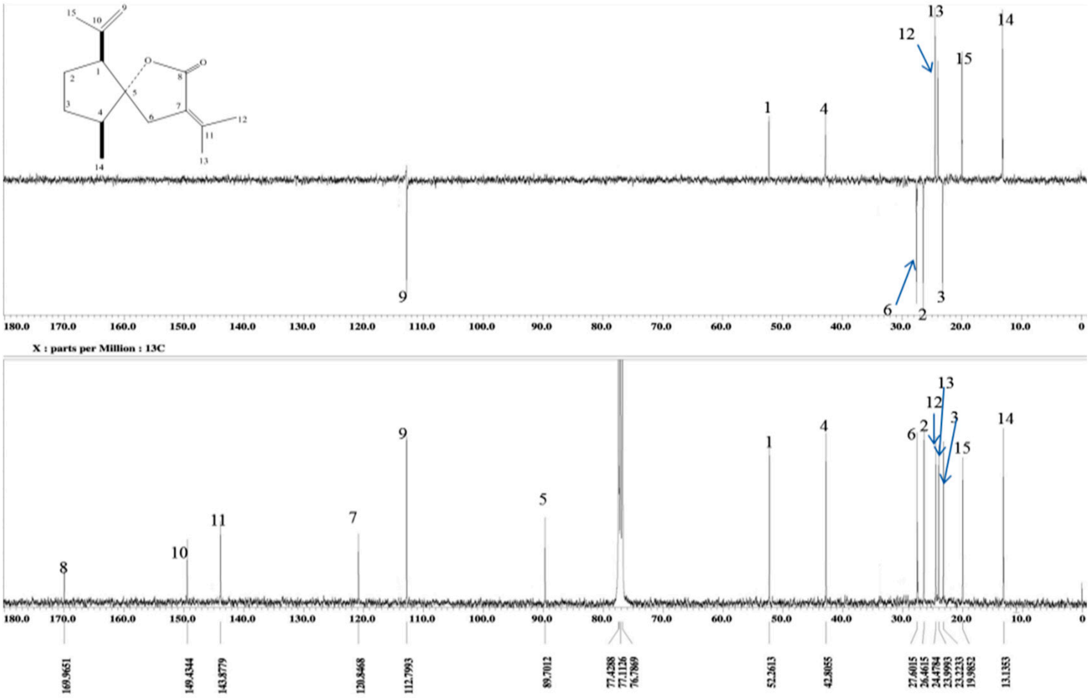

Figure S40. ^{13}C and DEPT spectra of Curcumanolide A (**20**) in $CDCl_3$. Curcumanolide A (**20**): ^{13}C NMR ($CDCl_3$, 100 MHz), δ: 19.9 (C-15), 13.1 (C-14), 23.9 (C-13), 24.4 (C-12), 143.8 (C-11), 149.4 (C-10), 112.7 (C-9), 169.9 (C-8), 120.8 (C-7), 27.6 (C-6), 89.7 (C-5), 42.8 (C-4), 23.22 (C-3), 26.5 (C-2), 52.3 (C-1).

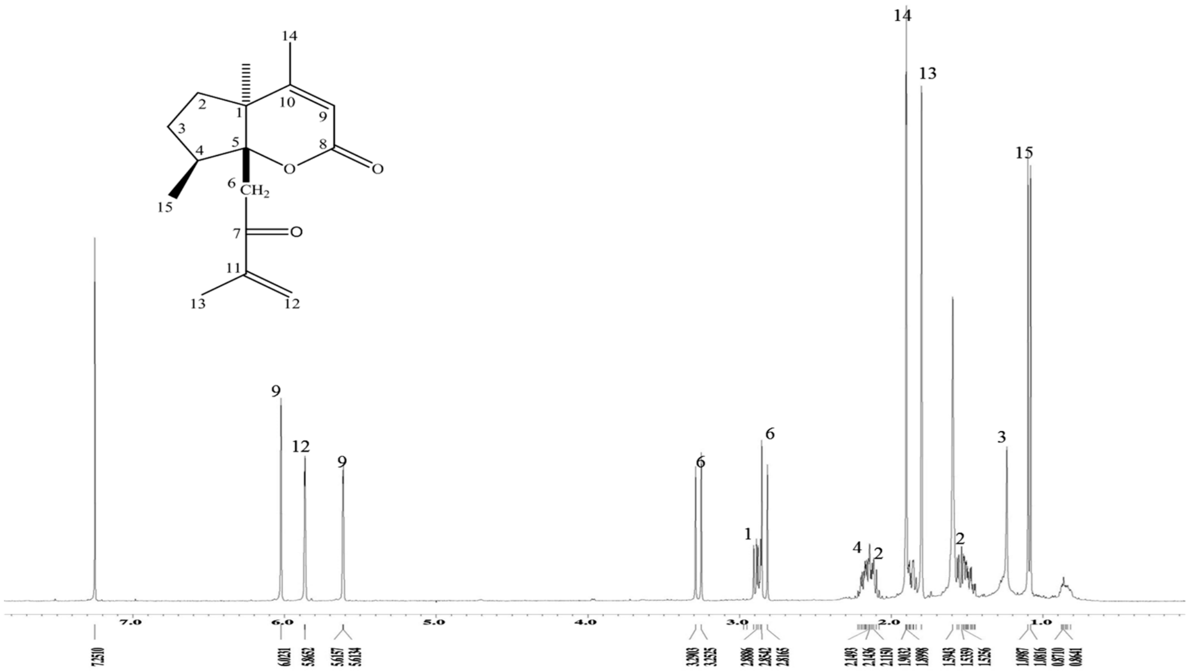

Figure S41. ^{1}H NMR spectrum of curcuzedoalide (**21**) in $CDCl_3$. Curcuzedoalide (**21**): ^{1}H NMR ($CDCl_3$, 400 MHz), δ: 1.09 (3H, *d*, *J* = 6.6 Hz, H-15), 1.91 (3H, *s*, H-14), 1.81 (3H, *s*, H-13), 5.88 (1H, *d*, *J* = 1.2 Hz, H-12$_b$), 6.03 (1H, *s*, H-12$_a$), 5.62 (1H, *d*, *J* = 1.2 Hz, H-9), 2.83 (1H, *d*, *J* = 14.0 Hz, H-6$_b$), 3.26 (1H, *d*, *J* = 14.0 Hz, H-6a), 2.11 (1H, *m*, H-4), 1.25, 1.91 (2H, *m*, H-3), 1.79, 2.17 (2H, *m*, H-2), 2.87 (1H, *dd*, *J* = 7.2, 10.0 Hz, H-1).

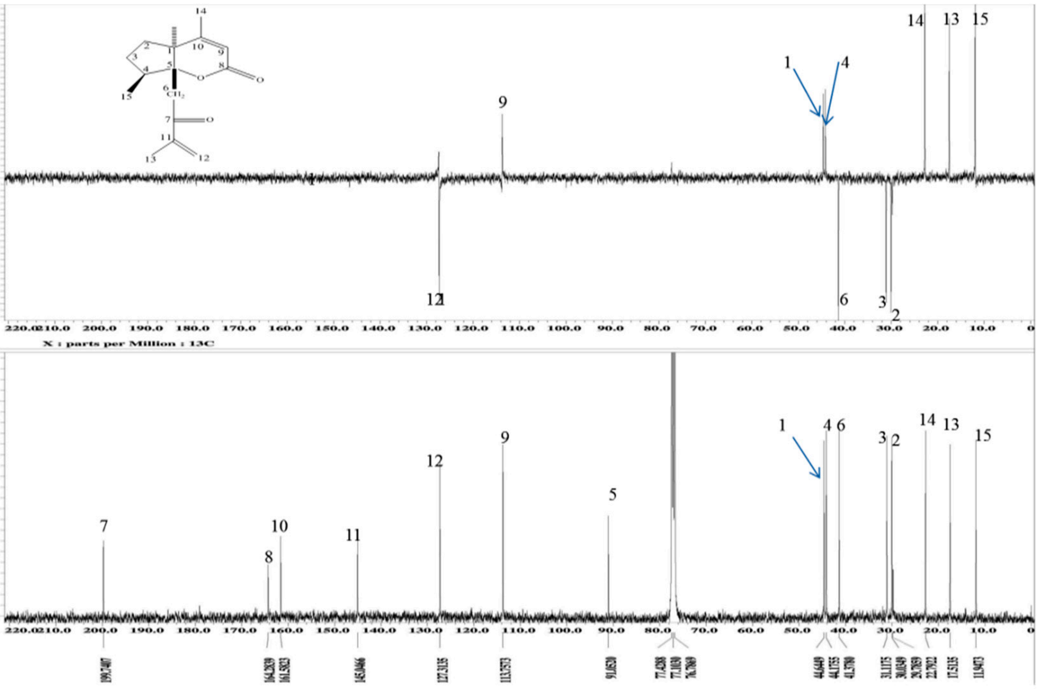

Figure S42. ^{13}C and DEPT spectra of curcuzedoalide (**21**) in $CDCl_3$. Curcuzedoalide (**21**): ^{13}C NMR ($CDCl_3$, 100 MHz), δ: 11.8 (C-15), 22.7 (C-14), 17.4 (C-13), 127.1 (C-12), 144.9 (C-11), 161.0 (C-10), 113.7 (C-9), 164.4 (C-8), 199.6 (C-7), 41.3 (C-6), 90.9 (C-5), 44.1 (C-4), 31.0 (C-3), 30.0 (C-2), 44.6 (C-1).
